# Developing a list of invasive alien species likely to threaten biodiversity and ecosystems in the European Union

**DOI:** 10.1111/gcb.14527

**Published:** 2018-12-12

**Authors:** Helen E. Roy, Sven Bacher, Franz Essl, Tim Adriaens, David C. Aldridge, John D. D. Bishop, Tim M. Blackburn, Etienne Branquart, Juliet Brodie, Carles Carboneras, Elizabeth J. Cottier-Cook, Gordon H. Copp, Hannah J. Dean, Jørgen Eilenberg, Belinda Gallardo, Mariana Garcia, Emili García‐Berthou, Piero Genovesi, Philip E. Hulme, Marc Kenis, Francis Kerckhof, Marianne Kettunen, Dan Minchin, Wolfgang Nentwig, Ana Nieto, Jan Pergl, Oliver L. Pescott, Jodey M. Peyton, Cristina Preda, Alain Roques, Steph L. Rorke, Riccardo Scalera, Stefan Schindler, Karsten Schönrogge, Jack Sewell, Wojciech Solarz, Alan J. A. Stewart, Elena Tricarico, Sonia Vanderhoeven, Gerard van der Velde, Montserrat Vilà, Christine A. Wood, Argyro Zenetos, Wolfgang Rabitsch

**Affiliations:** ^1^ Centre for Ecology & Hydrology Wallingford UK; ^2^ University of Fribourg Fribourg Switzerland; ^3^ Environment Agency Austria Vienna Austria; ^4^ Division of Conservation Biology, Vegetation Ecology and Landscape Ecology University Vienna Vienna Austria; ^5^ Research Institute for Nature and Forest (INBO) Brussels Belgium; ^6^ Department of Zoology University of Cambridge Cambridge UK; ^7^ The Laboratory The Marine Biological Association Plymouth UK; ^8^ University College London London UK; ^9^ Institute of Zoology Zoological Society of London London UK; ^10^ Invasive Species Unit Service Public de Wallonie Wallonia Belgium; ^11^ Natural History Museum London UK; ^12^ Royal Society for the Protection of Birds The Lodge Sandy Bedfordshire UK; ^13^ Scottish Association for Marine Science Scottish Marine Institute Oban UK; ^14^ Centre for Environment, Fisheries and Aquaculture Science Lowestoft UK; ^15^ Centre for Conservation Ecology Bournemouth University Poole UK; ^16^ Department of Plant and Environmental Sciences University of Copenhagen Denmark; ^17^ Pyrenean Institute of Ecology (IPE‐CSIC) Zaragoza Spain; ^18^ ISSG Rome Italy; ^19^ GRECO Institute of Aquatic Ecology University of Girona Girona Spain; ^20^ Institute for Environmental Protection and Research ISPRA, and Chair IUCN SSC Invasive Species Specialist Group Rome Italy; ^21^ Bio-Protection Research Centre Lincoln University Lincoln New Zealand; ^22^ CABI Delémont Switzerland; ^23^ Royal Belgian Institute of Natural Sciences (RBINS) Oostende Belgium; ^24^ Institute for European Environmental Policy London UK; ^25^ Marine Organism Investigations Marina Village, Ballina, Killaloe Co Clare Ireland; ^26^ University of Bern Bern Switzerland; ^27^ Institute of Botany The Czech Academy of Sciences Průhonice Czech Republic; ^28^ Ovidius University of Constanta Constanta Romania; ^29^ Institut National de la Recherche Agronomique Zoologie Forestière, UR 0633 Ardon Orleans Cedex 2 France; ^30^ Institute of Nature Conservation Polish Academy of Sciences Kraków Poland; ^31^ University of Sussex Brighton UK; ^32^ University of Florence Firenze Italy; ^33^ Belgian Biodiversity Platform Louizalaan, Brussels Belgium; ^34^ Institute for Water and Wetland Research Radboud University Nijmegen The Netherlands; ^35^ Naturalis Biodiversity Center Leiden The Netherlands; ^36^ Netherlands Centre of Expertise for Exotic Species (NEC‐E) Nijmegen The Netherlands; ^37^ Estación Biológica de Doñana, (EBD‐CSIC) Seville Spain; ^38^ Helenic Centre for Marine Research Anavyssos Greece

**Keywords:** biological invasions, consensus approach, environmental policy, impacts, introductions, prioritization, risk assessment

## Abstract

The European Union (EU) has recently published its first list of invasive alien species (IAS) of EU concern to which current legislation must apply. The list comprises species known to pose great threats to biodiversity and needs to be maintained and updated. Horizon scanning is seen as critical to identify the most threatening potential IAS that do not yet occur in Europe to be subsequently risk assessed for future listing. Accordingly, we present a systematic consensus horizon scanning procedure to derive a ranked list of potential IAS likely to arrive, establish, spread and have an impact on biodiversity in the region over the next decade. The approach is unique in the continental scale examined, the breadth of taxonomic groups and environments considered, and the methods and data sources used. International experts were brought together to address five broad thematic groups of potential IAS. For each thematic group the experts first independently assembled lists of potential IAS not yet established in the EU but potentially threatening biodiversity if introduced. Experts were asked to score the species within their thematic group for their separate likelihoods of i) arrival, ii) establishment, iii) spread, and iv) magnitude of the potential negative impact on biodiversity within the EU. Experts then convened for a 2‐day workshop applying consensus methods to compile a ranked list of potential IAS. From an initial working list of 329 species, a list of 66 species not yet established in the EU that were considered to be very high (8 species), high (40 species) or medium (18 species) risk species was derived. Here, we present these species highlighting the potential negative impacts and the most likely biogeographic regions to be affected by these potential IAS.

## Graphical abstract

We identified 66 species, that are currently absent from the EU, which pose a very high, high or medium threat to biodiversity and ecosystems. The species span a range of functional groups, with primary producers being numerically dominant. Escape from confinement is the pathway considered to be the most likely route of introduction for many species, particularly among plants and vertebrates.

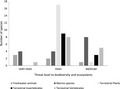

## 1 INTRODUCTION

There are currently more than 14,000 alien species recorded in Europe (EASIN Catalogue, https://easin.jrc.ec.europa.eu/) with more than half originating from outside EU territories, while the remainder have originated within parts of the EU and subsequently invaded others. Their numbers are rapidly increasing (Seebens et al., 2017), and in some cases so is their rate of spread (Roques et al., 2016). A number of alien species cause serious problems for the environment and society (Vilà et al., 2010) and these are termed invasive alien species (IAS) (European Union, 2014). The European Commission has addressed the threat of IAS in their Regulation 1143/2014; at the heart of the regulation is the development of a list of IAS of EU concern, with an explicit focus on potential future invaders, excluding some microorganisms,^1^ 1The Regulation does not apply to:

- Species changing their natural range without human intervention, in response to changing ecological conditions and climate change;
- Genetically modified organisms as defined in point 2 of Article 2 of Directive 2001/18/EC;
- Pathogens that cause animal diseases; for the purpose of this Regulation, animal disease means the occurrence of infections and infestations in animals, caused by one or more pathogens transmissible to animals or to humans;
- Harmful organisms listed in Annex I or Annex II to Directive 2000/29/EC, and harmful organisms for which measures have been adopted in accordance with Article 16(3) of that Directive;
- Species listed in Annex IV to Regulation (EC) No 708/2007 when used in aquaculture;
- Micro‐organisms manufactured or imported for use in plant protection products already authorized or for which an assessment is ongoing under Regulation (EC) No 1107/2009; or
- Micro‐organisms manufactured or imported for use in biocidal products already authorized or for which an assessment is ongoing under Regulation (EU) No 528/2012.

that will be targeted for action (European Union, 2014; Genovesi, Carboneras, Vilà, & Walton, 2015). Thus, the identification of likely future IAS is pivotal for implementing this regulation. Here, we present a horizon scanning approach to identify likely future IAS to inform the list of IAS of EU concern.

Horizon scanning can be defined as a systematic examination of potential threats and opportunities, within a given context, and likely future developments which are at the margin of current thinking and planning (Food Standards Agency, 2018). There are a number of approaches that could be adopted for horizon scanning (Supporting information S1: Overview of approaches horizon scanning methods) with varying strengths and weaknesses depending on the context (Sutherland & Woodroof, 2009). Horizon scanning usually follows a structured process of simplification and reduction from a large set of data to a prioritized subset categorized by the most important and relevant data. A series of recent papers have provided convincing arguments that horizon scanning should play a more prominent role in environmental and conservation practice (Copp et al., 2016; Cowx, Angelopoulos, Nunn, Britton, & Copp, 2009; IPCC 2005; Ricciardi et al., 2017; Sutherland & Woodroof, 2009; Van Wilgen & Richardson, 2012) including as a tool for informing policies on IAS, particularly through preventing arrival (Copp, Templeton, & Gozlan, 2007; Shine et al., 2010).

There have been a number of horizons scanning exercises for IAS in Europe, but these have usually involved one or few taxonomic groups, such as plants (Andreu & Vilà, 2010; Thomas, 2010) or animals (Parrott et al., 2009), or distinct environments such as freshwater (Gallardo & Aldridge, 2013), specific countries (Matthews et al., 2014; Roy, Peyton et al., 2014; Matthews et al., 2017), or regions (NOBANIS 2015; Gallardo et al., 2016). Most of these approaches have relied on information from the literature coupled with impact assessment frameworks (Parrott et al., 2009; Thomas, 2010) or modelling approaches (Gallardo & Aldridge, 2013). It has been noted that wildlife diseases are lacking within horizon scanning exercises and that there is a need to address this imbalance (Roy et al., 2017).

A horizon scanning exercise for Great Britain was carried out in 2013 and illustrates the merits of using a combination of approaches and concluding with a consensus workshop to create a ranked list of IAS (all plant and animal taxa, excluding microorganisms, across all environments) that are likely to arrive, establish and have an impact on native biodiversity within the following 10 years (Roy, Peyton et al., 2014). Within 2 years of publication of this list, seven of the species ranked within the top ten had been newly recorded within Great Britain. Most notably, the quagga mussel, *Dreissena rostriformis bugensis*, which was given the maximum scores for risk of arrival, establishment and impact and accordingly ranked in the top position, was reported in October 2014 (Aldridge, Ho, & Froufe, 2014).

There are considerable strengths to such consensus methods, particularly when information is limited, but it is important to be aware that opinion is not knowledge (Banks, Wright, Maclean, Hann, & Rehfisch, 2008). Indeed, it is critical that consensus methods, in which experts are engaged, adequately address issues with respect to accuracy and judgement to reduce the effects of potential bias (Sutherland & Burgman, 2015; Garnas et al., 2016). Discussions through consensus approaches, where not just scores are communicated, but also the insights that led to them, can reduce levels of uncertainty. Uncertainty is inherent when dealing with data deficiency (e.g. insufficient information on species) and ambiguity in terminology, which is a problem in invasion ecology, particularly between experts from different taxonomic groups (Essl et al., 2016). Indicating the perceived level of confidence of the assessments, and documenting the discussions behind the agreed level (or score) of uncertainty, is therefore considered crucial in communicating the outcome of the exercise to a wider scientific or public audience. During the consensus building process, lack of evidence or contradictory information can easily be tracked and discussed. Therefore, the method is particularly useful to integrate scarce information available for many potential alien species (Vanderhoeven et al., 2017).

Here, we present a consensus approach which was adopted for the first EU‐wide horizon scan for future IAS not native to any parts of Europe with the potential to threaten European biodiversity. The EU‐wide horizon scan was part of a study funded by the European Commission for prioritization of IAS (Roy, Peyton et al., 2014). This study is unique in the continental scale examined but also the breadth of taxonomic groups and environments considered. The proposed list provides a basis for prioritizing full risk assessments of species not yet established in the EU in order to comprehensively evaluate the threat posed by these species to EU biodiversity. The study may also serve as a model for future horizon scanning projects of similar thematic or geographic scope.

## 2 MATERIALS AND METHODS

**Figure 1:**
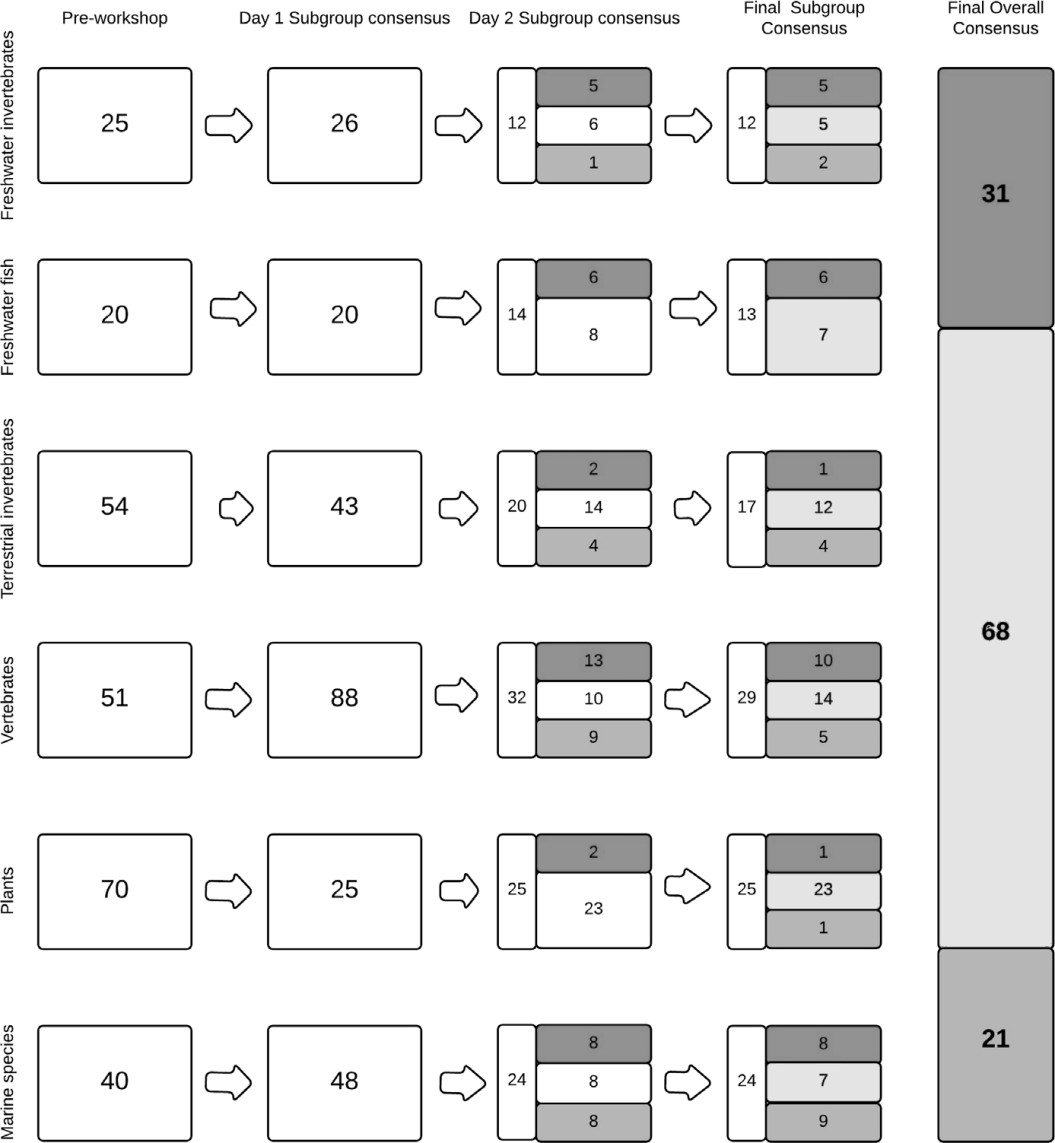
Number of species for each thematic subgroup (Freshwater invertebrates, Freshwater fish, Terrestrial invertebrates, Vertebrates, Plants and Marine species) at different stages of the horizon scanning process (preworkshop, Day 1 Subgroup Consensus, Day 2 Subgroup Consensus, Final Subgroup consensus and Final Overall Consensus). Note the Final Overall Consensus includes species that have a limited distribution within the EU and those that are considered absent from the EU; for the latter category there was a total of 66 species (with 18, 40 and 8 species considered to represent medium, high and very high threat respectively). White = unranked, dark grey = very high, light grey = high, mid grey = medium priority for risk assessment

We used an adapted version of the consensus method (Sutherland, Fleishman, Mascia, Pretty, & Rudd, 2011; Roy, Peyton et al., 2014) for a horizon scanning approach to derive a ranked list of species to be risk assessed, hence to be further considered to derive a list of potential IAS with high impact on biodiversity (Figure 1). It is important to note that the process was undertaken in the framework of the EU Regulation 1143/2014 on IAS and accordingly the approach (and particularly scope) was in part determined by this context (Roy et al. 2014). The approach involved a sequence of critical steps:

### 2.1 Step 1. Establishment of thematic groups

Five broad thematic groups (plants, terrestrial invertebrates, marine species, freshwater invertebrates and vertebrates) of IAS and associated experts based on taxonomy and major environments were established (Supporting information S2). The experts were selected to provide representation across Europe and ensure sufficient knowledge across taxonomic groups and environments. Group size ranged between six to nine experts and contained two co‐leaders who agreed to coordinate and record activities and discussion between group members before, during and after the workshop.

### 2.2 Step 2. Compilation of preliminary lists of potential IAS

Each thematic group was asked to assemble preliminary lists of potential IAS that they considered to constitute the highest risk with respect to the likelihood of arrival, establishment, spread and the magnitude of their potential negative impact on biodiversity and ecosystem services, within the EU region over the next 10 years. It was expected that each thematic group would derive these lists from a combination of systematic literature searches (including academic journals, risk assessments, reports, authoritative websites and other “grey” literature), querying of IAS databases (Supporting information S3) and their own expert knowledge. As expected, the approaches adopted by each thematic group differed slightly with respect to methods followed to derive the preliminary lists because of the diverse nature of the taxonomic groups and variation in the sources of information available (details given in Supporting information S4). However, initially all experts worked independently to provide lists of potential IAS for consideration by the entire group at a later stage.

The geographic scope of the search for potential IAS was worldwide. It was clearly stated that the lists should only include species alien to the EU, including the Macaronesian islands, but excluding other EU outermost regions, acknowledging that the EU does not encompass the entire European continent. A potential, but not exhaustive, list of search criteria included alien species that:

1. Are absent in the EU
2. Are present in countries close to or sharing a border with the EU
3. Are present in areas of the world that are climatically matched to the study region (using the Köppen‐Geiger climate zones as reference)
4. Have documented histories of invasion and causing undesirable impacts in other regions worldwide
5. Are traded within the EU or are present in areas that have strong trade or travel connections with the EU and where there is a recognized potential pathway for arrival
6. Are present in captivity including zoological parks, aquaculture facilities and glass houses.

The temporal scope of the horizon scanning exercise was that only species likely to arrive in the next 10 years within the EU should be included. This temporal limit had important consequences, because it limited the relevance of, for instance, long‐term climate change projections.

A simplified framework was developed following the workshop. It was decided to focus on five climatic zones based on the biogeographic regions of Europe as defined by the European Environment Agency (EEA, see http://ec.europa.eu/environment/nature/natura2000/biogeog_regions/). A correspondence with Köppen‐Geiger climate zones (Kottek, Grieser, Beck, Rudolf, & Rubel, 2006) was provided to allow extrapolation of species establishment potential based on the species distribution in other continents. For marine species (all species living within the sea), the framework was modified by adding the Baltic Sea, Mediterranean and Black Seas.

The scope of the exercise was further refined based on a number of exclusions including those already stated above:

1. Species that arrive from their native range by natural spread/dispersal without human intervention in response to changing ecological conditions or climate change
2. Parasites that cause animal diseases (including to wildlife)
3. Species or taxonomic groups that are regulated under EU legislations other than the EU Regulation 1143/2014 on IAS (e.g. EU Plant Health Legislation – Directive 2000/29/EC or EU regulation on the use of alien species in aquaculture ‐ Regulation (EC) No 708/2007)
4. Microorganisms and fungi
5. Species having adverse impacts only in productive sectors (such as agriculture, horticulture, timber) or on human health and wellbeing, unless these impacts are in addition to separate impacts on native biodiversity (in which case, these additional impacts were noted, but not used as primary selection criteria).

The consultation between experts was completed both through e‐mail discussions in advance of the workshop (over 6 weeks) and through the workshop breakout groups. Co‐leaders of each of the thematic groups collated the lists of IAS received from the experts within their group into a single provisional list.

### 2.3 Step 3: Scoring of species

Experts were asked to independently score each species within their thematic group for their separate likelihoods of: (a) arrival, (b) establishment, (c) spread, and (d) magnitude of the potential negative impact on biodiversity within the EU. A 5‐point scale from 1 = very low to 5 = very high (Blackburn et al., 2014) was adopted to achieve an appropriate balance between accuracy and resolution. The scores from each expert within each thematic group were then compiled and discussions within the thematic groups (at the workshop) led to an overall agreed impact and confidence score for each species with respect to likelihoods of: (a) arrival, (b) establishment, (c) spread, and (d) impact on biodiversity. Further guidance on species scoring is given below.

Scores for the likelihood of arrival were based on a consideration of several relevant factors, including: previous history of invasion by the species in other regions; the existence of a plausible introduction pathway; qualitative consideration of volume and frequency of trade and travel between the existing range of the species and the EU. A score of 1 denoted that the species was considered unlikely to arrive in the EU within the chosen timeframe. A score of 5 was used to denote near‐certain, arrival. In the case of species already in the EU (such as those held commonly in captivity or planted in gardens), the likelihood of arrival was agreed to be the top category of 5.

Having arrived, the probability of a species establishing a self‐sustaining population in the wild will depend on the ecological properties of both the species itself and the community that it is invading (Leung et al., 2012). Scores therefore reflected life‐history characteristics including reproductive rate and ecological features such as tolerance of a broad range of environmental conditions or availability of food supply in the introduced range. Scores for likelihood of spread were primarily determined by the dispersal ability of the species, both natural and human‐assisted, and its history and speed of spread in other regions where invasive.

Experts were asked to score the magnitude of impact on biodiversity and ecosystem functions related to ecosystem services, and the likelihood of colonization of habitats of high conservation value (as defined by the EU Habitats Directive). Furthermore, information was requested on the mechanisms through which each IAS could impact biodiversity and ecosystem functions (Supporting information S5).

The impact scoring system was modified from the ISEIA protocol (Branquart, Verreycken, Vanderhoeven, & Van Rossum, 2009; De Groot, Alkemade, Braat, Hein, & Willemen, 2010), the GB NNRA (Booy, White, & Wade, 2006) and the proposed unified framework for environmental impacts ‐ EICAT (Blackburn et al., 2014; Hawkins et al., 2015). The descriptors of the impact scoring system are provided in Supporting information S5. Confidence levels (Supporting information S5) were attributed to each score to help focus discussions and refine the list of species but were not used formally within the consensus building (across all thematic groups). Therefore, confidence scores are not reported here but did prove useful in guiding discussion within some thematic groups.

While acknowledging that the scores were only for guidance on ranking and not to be used as absolute, an overall risk score for each species was calculated as the product of the individual scores for arrival, establishment, spread and impact on biodiversity as proposed in the Harmonia+ protocol. With a 4‐criterion, 5‐point scoring system, this produces a maximum score of 625. The individual completed spreadsheets from each expert were then returned to group leaders for collation. The objective was to reach broad consensus on the scores within each group in advance of the workshop. This was achieved through e‐mail and Skype discussions between group members but the workshop provided an opportunity for further refinement by the experts.

### 2.4 Step 4: Expert (consensus) workshop

The aims of the 2‐day workshop were clearly outlined; then an overview of the IAS selected by each thematic group was presented. These thematic group presentations were particularly important because they informed the other participants of the range of species and their life‐histories within each group, enabling subsequent review and moderation of the scores within the breakout sessions for each thematic group. During the breakout session, participants were requested to add or remove species in the light of new evidence (either discovered just prior to the workshop or following reflection from the preceding workshop presentations and discussions), to justify and moderate scores through discussion and to consider levels of confidence attached to scores. The thematic groups were asked to restrict their lists to a total of 20–30 top‐ranked species. The emphasis at this stage was to use the scores as guidance for informing the subsequent consensus‐building component of the horizon scanning approach and deriving a ranked list rather than as a component of a full impact assessment.

All the species lists from across the thematic groups were collated into a single list. At this stage there were 249 species listed (Supporting information S6). Experts were invited to justify their scores in comparison to those of other groups, to increase the alignment of results among groups through a further round of review and moderation of the lists. The lists (Supporting information S7) from each thematic group were again combined to produce a list of 120 species. The process of sequential reduction in number of IAS prioritised for each thematic group is summarized in Figure 1.

All participants were then invited to review, consider and refine the rankings of all species through plenary discussion. Leaders of each thematic group were again asked to justify to the other workshop participants the scores for their top‐scoring species and to respond to queries or objections from members of other thematic groups. It proved to be challenging, but very fruitful, to discuss rankings across thematic groups. Changes to overall rankings for individual species were made only after hearing the evidence from appropriate experts, full discussion and, if needed, majority voting. The end result was an agreed ranked list of potential IAS derived through discussion and broad consensus that were considered to represent a medium, high or very high probability of arrival, establishment, spread and magnitude of impact on biodiversity and ecosystem services (Figure 1).

### 2.5 Step 5: Post workshop compilation of information on species

Following the workshop, information was gathered by the experts within the thematic groups on the likely pathways of arrival (CBD 2014), using published classifications (Supporting information S8). Additionally the biogeographic regions in the EU likely to be most threatened by each species were documented.

### 2.6 Statistical analysis

To analyse frequencies among thematic groups in relation to threat, pathways of arrival and membership of functional groups we used Chi‐squared tests. Count data of biogeographic regions under threat were analysed by generalized linear models with quasi‐poisson distibutions. The latter was used to account for underdispersion in the residuals (Crawley, 2012).

## 3 RESULTS

**Figure 2:**
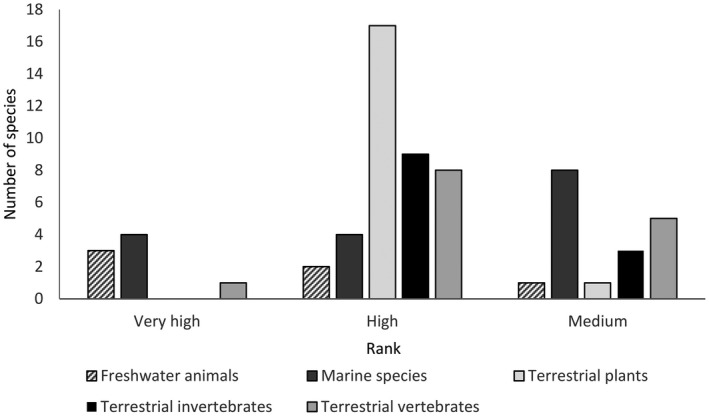
Number of species absent from Europe (n = 66) that were considered to have a very high, high or medium probability of arrival, establishment, spread and magnitude of impact on biodiversity and ecosystem services across thematic subgroups (Freshwater invertebrates, Freshwater fish, Terrestrial invertebrates, Vertebrates, Plants and Marine species)

Of the 329 species considered, a total of 66 marine, terrestrial and freshwater species were identified as having medium (18 species), high (40 species) or very high (8 species) overall threat (Table 1; Figure 2). All workshop participants agreed that the list represented the outcome of the consensus approach.

**Table 1:** List of 66 potential IAS in the EU with very high (8 species; no fill), high (40; dark grey fill) or medium (18; normal text) perceived overall threat

								Impact type											
Species Name	Common Name	Thematic Group	Functional Group	CBD Pathway	Native range	Biogeographic regions threatened	Combined Risk Score	Competition	Predation	Hybridization	Disease Transmission	Poisoning or toxicity	Bio‐fouling	Grazing/herbivory/browsing	Interactions with invasive alien species	Nutrient cycling	Physical modification	Natural succession	Disruption to food webs
*Channa argus*	Northern snakehead	Freshwater fish	Predator	Escape, Release	At	MAC, MED, ATL, CON, STE	383	X	X		X								X
*Limnoperna fortunei*	Golden mussel	Freshwater invertebrate	Filter feeder	Stowaway, Corridor, Unaided	As	MED, ATL, CON, STE	500	X					X	X	X	X	X		X
*Orconectes rusticus*	Rusty crayfish	Freshwater invertebrate	Omnivore	Escape, Release, Stowaway, Corridor, Unaided	NAm	MED, ATL, CON, STE	500	X	X		X			X	X	X	X		X
*Plotosus lineatus*	Striped eel catfish	Marine	Predator	Unaided	WIP, TeNWP, CIP, TeAu	MED, MAC	456	X	X			X							X
*Codium parvulum*	A green alga	Marine	Primary producer	Unaided	WIP	MED, MAC	400	X					X		X	X	X		
*Crepidula onyx*	Onyx slippersnail	Marine	Filter feeder	Escape, Stowaway	TrEP	ATL, MED, MAC	240	X					X			X	X		
*Mytilopsis sallei*	Black striped mussel	Marine	Filter filter	Stowaway	TrWA	MED, MAC, ATL, BAL, BLK	216	X	** **				X			X	X	X	X
*Sciurus niger*	Fox squirrel	Vertebrate	Herbivore	Escape, Release	NAm	ATL, MED, CON	405	X	X					X	X		X		X
*Morone americana*	White perch	Freshwater fish	Predator	Escape	NAm	MAC, MED, ATL, CON, STE	221		X		X								X
*Albizia lebbeck*	Indian siris	Plant	Primary producer	Escape	At	MAC, ATL, MED	300	X								X	X	X	
*Celastrus orbiculatus*	Oriental bittersweet	Plant	Primary producer	Escape	As	ATL, BOR, CON, (MED)	500												
*Chromolaena odorata*	Siam weed	Plant	Primary producer	Contaminant	SAm	MAC, MED	320	X			X						X	X	
*Cinnamomum camphora*	Camphor tree	Plant	Primary producer	Escape	As, At	MAC, ATL	400	X				X					X		X
*Clematis terniflora*	Leather leaf clematis	Plant	Primary producer	Escape	As, At	MAC, ATL	400	X									X		
*Cortaderia jubata*	Purple pampas grass	Plant	Primary producer	Escape	SAm	ATL, MAC, MED	500	X									X	X	
*Cryptostegia grandiflora*	Rubber vine	Plant	Primary producer	Escape	SAm	MAC, ATL, MED	320	X				X	X				X	X	
*Gymnocoronis spilanthoides*	Senegal tea	Plant	Primary producer	Escape	As, SAm	MAC, (MED)	625	X								X	X	X	
*Lespedeza juncea* ssp. *sericea (= L. cuneata)*	Chinese lespedeza	Plant	Primary producer	Escape	As, Aus	ATL, CON, MAC, MED	500	X				X					X		
*Lonicera maackii*	Amur honeysuckle	Plant	Primary producer	Escape, Release	As	ATL, CON, MAC, MED	500	X									X		X
*Lonicera morrowii*	Morrow's honeysuckle	Plant	Primary producer	Escape, Release	As	ATL, CON, MAC, MED	500	X									X		X
*Lygodium japonicum*	Japanese climbing fern	Plant	Primary producer	Escape	At	MAC, (MED)	625	X									X	X	
*Microstegium vimineum*	Nepalese browntop	Plant	Primary producer	Contaminant	As	(ATL), CON, MAC, MED	500	X								X	X		X
*Prosopis juliflora*	Prosopis	Plant	Primary producer	Contaminant, Escape	SAm	ATL, MAC, MED	500	X							X	X	X	X	
*Prunus campanulata*	Bell flower cherry	Plant	Primary producer	Escape	As	ATL, MAC	500	X									X		
*Rubus rosifolius*	Roseleaf raspberry	Plant	Primary producer	Escape	At, Aus	MAC	500	X		X					X		X	X	X
*Triadica sebifera (Sapium sebiferum)*	Chinese tallowtree	Plant	Primary producer	Escape	As	MAC, MED	500	X								X	X	X	X
*Acanthophora spicifera*	A red alga	Marine	Primary producer	Stowaway	TrWA	MED, MAC	192	X					X			X	X	X	
*Gammarus fasciatus*	Freshwater shrimp	Freshwater	Omnivore	Escape, Contaminant, Corridor, Stowaway	NAm	MED, ATL, CON, STE	108	X	X					X	X	X		X	X
*Perna viridis*	Asian green mussel	Marine	Filter feeder	Stowaway	TeNWP	MED, MAC, ATL	192	X				X	X		X	X	X		
*Potamocorbula amurensis*	Asian basket clam	Marine	Filter feeder	Stowaway	TeNWP	MED, MAC, ATL, BLK, BAL	180	X	X					X	X	X	X	X	X
*Symplegma reptans*	Pillow‐like tunicate	Marine	Filter feeder	Stowaway	TeNWP	MED, MAC, ATL, BLK	192	X					X			X	X		
*Aeolesthes sarta*	City longhorn beetle, Qetta borer	Terrestrial invertebrate	Herbivore	Contaminant	As	MED, ATL, CON, STE, BOR	99	X						X	X	X	X	X	X
*Amynthas agrestis*	Crazy snake worm	Terrestrial invertebrate	Detritivore	Contaminant, Stowaway	As?	ATL, CON	129	X							X	X	X	X	X
*Pachycondyla chinensis*	Asian needle ant	Terrestrial invertebrate	Omnivore	Contaminant	As	MED, ATL, CON, STE, MAC	175	X	X						X	X	X	X	X
*Sirex ermak*	Blue‐black horntail	Terrestrial invertebrate	Herbivore	Contaminant, Escape	As	CON, STE, BOR	111	X			X			X		X	X	X	X
*Solenopsis geminata*	Fire ant	Terrestrial invertebrate	Omnivore	Contaminant	NAm/SAm	MAC, MED, ATL?, CON?, STE	160	X	X			X			X	X	X	X	X
*Solenopsis invicta*	Red imported fire ant	Terrestrial invertebrate	Omnivore	Contaminant	SAm	MAC, MED	160	X	X			X		X	X	X	X	X	X
*Solenopsis richteri*	Black imported fire ant	Terrestrial invertebrate	Omnivore	Contaminant	SAm	MAC, MED, ATL?, CON?, STE	128	X	X			X		X	X	X	X	X	X
*Tetropium gracilicorne*	Fine‐horned spruce beetle	Terrestrial invertebrate	Herbivore	Contaminant	As	ATL, CON, STE, BOR	128	X						X	X	X	X	X	X
*Vespula pensylvanica*	Western yellowjacket	Terrestrial invertebrate	Omnivore	Contaminant	NAm	MAC?, MED, ATL, CON, STE, BOR?	99	X	X			X			X	X			X
*Bison bison*	American bison	Vertebrate	Herbivore	Release	NAm	CON	338	X		X	X			X					
*Boiga irregularis*	Brown tree snake	Vertebrate	Predator	Escape, Release, Stowaway	Aus	MED, MAC	280	X	X			X			X				X
*Cynops pyrrhogaster*	Japanese fire‐bellied salamander	Vertebrate	Omnivore	Escape	As	CON	354	X	X	?	X							X	X
*Eleutherodactylus coqui*	Common coquí	Vertebrate	Predator	Escape, Contaminant, Stowaway	NAm	MED, MAC	252		X	X									
*Eleutherodactylus planirostris*	Greenhouse frog	Vertebrate	Predator	Escape, Stowaway	NAm	MED, MAC	288	X	X		X								
*Hemidactylus frenatus*	Common house gecko	Vertebrate	Predator	Escape, Stowaway	Aus	ATL, MED, CON	320	X	X										
*Rhinella marina*	Cane toad	Vertebrate	Omnivore	Contaminant, Escape, Release,	SAm	MED, MAC	280	X	X			X			X	X			X
*Trichosurus vulpecula*	Brushtail possum	Vertebrate	Omnivore	Escape	Aus	ATL, MED, CON, MAC	304	X	X		X			X					
*Daphnia lumholtzi*		Freshwater invertebrate	Filter feeder	Contaminant, Corridor, Stowaway, Unaided	As, At, Aus	MAC, MED, CON, STE	96	X						X	X	X		X	X
*Pinus patula*	Mexican weeping pine	Plant	Primary producer	Escape	NAm	MAC, ATL	300	X									X	X	
*Ascidia sydneiensis*	Green tube tunicate	Marine	Filter feeder	Contaminant, Stowaway	WIP, CIP	MED, MAC, ATL	108	X					X			X	X		
*Balanus glandula*	Acorn barnacle	Marine	Filter feeder	Stowaway	TeNEP	ATL, BAL	108	X					X		X		X	X	X
*Ciona savignyi*	Pacific transparent tunicate	Marine	Filter feeder	Escape, Stowaway	TeNEP, Teswa, TeAu	ATL, BLK, BAL, MED, MAC	144	X					X			X	X		
*Dictyosphaeria cavernosa*	Green bubble weed	Marine	Primary producer	Stowaway	WIP	MED, MAC	108	X					X			X	X	X	X
*Didemnum perlucidum*	A colonial tunicate	Marine	Filter feeder	Contaminant, Stowaway	Unknown	MED, MAC	128	X					X					X	X
*Dorvillea similis*	A polychaete worm	Marine	Detritivore	Stowaway, Unaided	WIP, CIP	MED, MAC	150												
*Rhodosoma turcicum*	A unitary tunicate	Marine	Filter feeder	Stowaway	WIP, CIP, TeNWP, TrWA	MED, MAC, ATL	162	X					X			X	X		
*Zostera japonica*	Dwarf eelgrass	Marine	Primary Producer	Contaminant	TeNWP	MED, MAC, ATL, BLK, BAL	108	X					X			X	X	X	X
*Agrilus auroguttatus*	Gold spotted oak borer	Terrestrial invertebrate	Herbivore	Contaminant, Stowaway	Nam	MED	81	X						X	X	X	X	X	X
*Dendrolimus superans*	White‐lined silk moth	Terrestrial invertebrate	Herbivore	Contaminant	As	CON, STE, BOR	128	X						X		X	X	X	X
*Platypus quercivorus*	Oak ambrosia beetle	Terrestrial invertebrate	Herbivore	Contaminant, Stowaway	As	MAC, MED, ATL, CON, STE	97	X			X			X	X	X	X	X	X
*Boa constrictor*	Boa constrictor	Vertebrate	Predator	Escape	SAm	MED	263	X	X							X			X
*Gymnorhina tibicen*	Australian magpie	Vertebrate	Omnivore	Escape	Aus	ATL, MED, CON	225												
*Python molurus*	Indian rock python	Vertebrate	Predator	Escape	At	MED	263	X	X							X			X
*Quelea quelea*	Red billed quelea	Vertebrate	Omnivore	Escape,Release	Afr	MED,CON	252												
*Tamiasciurus hudsonicus*	American red squirrel	Vertebrate	Herbivore	Escape	NAm	BOR, ATL, CON	244	X	X		X				X		X		

Notes

**Figure 3:**
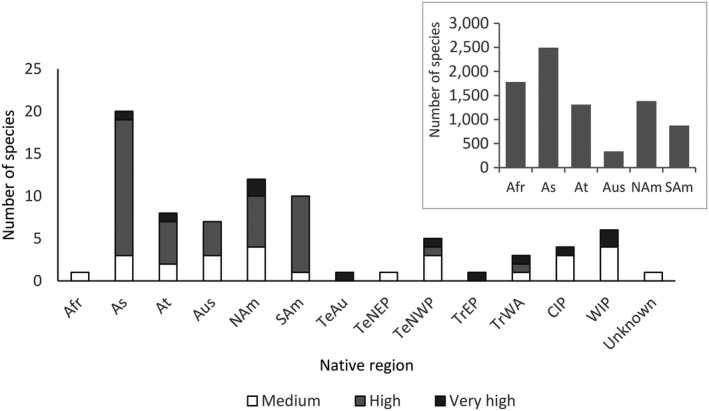
Native range of the species absent from Europe (*n* = 66) considered to have at least a medium probability of arrival, establishment, spread and magnitude of impact on biodiversity and ecosystem services. The insert shows the native regions of established terrestrial and freshwater alien species in Europe (*n* = 6,224, from DAISIE, 2009). Note that species can occur in more than one region. Terrestrial and Freshwater (Continental TDWG categories): Afr = Africa; As = Asia Temperate; At = Asia Tropical; Aus = Australasia; NAm = North America; SAm = South America. Marine (Spalding et al., 2007): TeAu = Temperate Australasia; TeNWA = Temperate NW Atlantic; TeNWP = Temperate NW Pacific; TeSAf = Temperate Southern Africa; TrEA = Tropical Eastern Atlantic; TrEP = Tropical Eastern Pacific; TrWA = Tropical Western Atlantic; CIP = Central Indo‐Pacific; WIP = Western Indo‐Pacific

The pathway information is as recommended by the (CBD 2014). Codes for Native Range are given in the legend for Figure 3 and for Biogeographic Regions in the legend for Figure 7. Note that the combined risk score was used for guiding the discussions between experts across thematic groups and **does not** relate strictly to the final rank of the species within the list. Likely impact type is indicated by “X” in the relevant columns.

It was notable that none of the plants or terrestrial invertebrates were ranked within the very high category, but 17 plants and 9 terrestrial invertebrates were considered as posing a high probability of arrival, establishment, spread and magnitude of impact on biodiversity and ecosystem services, and thus categorized as high impact. Of the 66 species identified, plants were considered to pose a higher than average and marine species a lower than average threat (χ^2^ = 9.32, *df* = 5, *p *< 0.05).

### 3.1 Native range

The highest proportions of the species identified through the horizon scanning have native ranges in Asia, North America and South America (Figure 3), which more or less mirrors the native ranges of currently established terrestrial and freshwater alien species in Europe (DAISIE, 2009). Species with native ranges in Africa are less represented in the pool of potential future invaders. The marine species are likely to originate from a range of geographic regions.

### 3.2 Pathways of arrival

**Figure 4:**
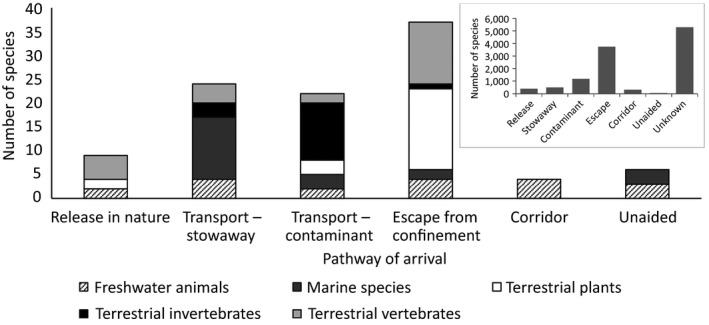
Number of species absent from Europe (n = 66) considered to have at least a medium probability of arrival, establishment, spread and magnitude of impact on biodiversity and ecosystem services and their anticipated pathways of arrival across thematic subgroups (Freshwater invertebrates, Freshwater fish, Terrestrial invertebrates, Vertebrates, Plants and Marine species). The pathway classification follows (CBD, 2014). The insert shows the frequencies of known pathways of currently established aliens in Europe as taken from DAISIE (2009) (n = 6,224)

Many of the species listed were anticipated to arrive along multiple pathways (Table 1; Figure 4), but it was apparent that escape from confinement was particularly relevant to plants and vertebrates, whereas aquatic species were considered to be most likely to arrive as stowaway or via shipping, and terrestrial invertebrates as contaminants (Figure 4). While the escape pathway was also the most important one in the past for currently established aliens in Europe (60% of all known pathways (*n* = 6,224, DAISIE, 2009), the importance of the stowaway pathways was considered likely to increase for future invaders from currently 8.1% (DAISIE, 2009) to 24% (Figure 4).

**Figure 5:**
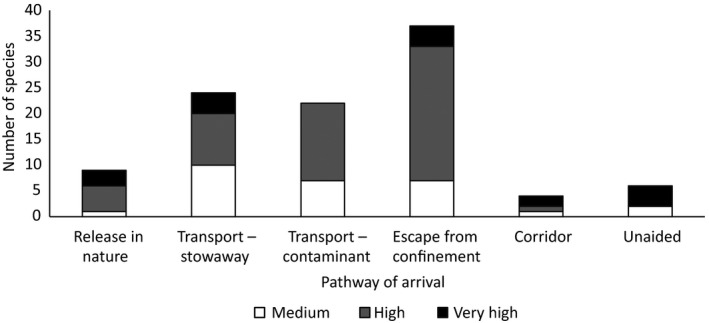
Number of species absent from Europe (n = 66) considered to have a very high, high or medium probability of arrival, establishment, spread and magnitude of impact on biodiversity and ecosystem services and their anticipated pathways of arrival. Note that species can arrive via multiple pathways. The pathway classification follows (CBD, 2014)

Our results do not indicate that there is any one statistically significant pathway through which high risk IAS are expected to enter Europe in future (χ^2^ = 5.3, *df* = 5, *p* = 0.38; Figure 5).

### 3.3 Functional groups

**Figure 6:**
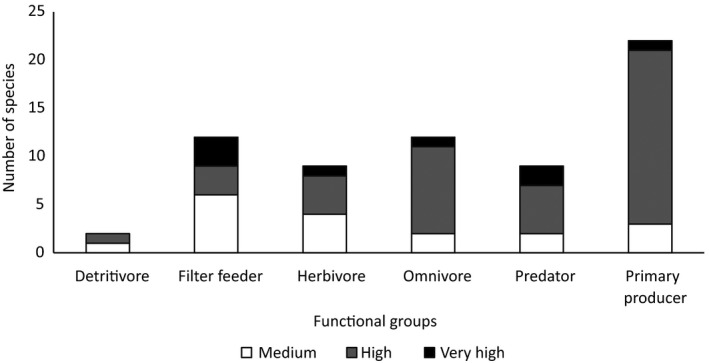
Number of species, absent from Europe (n = 66), within different functional groups considered to have at medium, high or very high probability of arrival, establishment, spread and impact on biodiversity and ecosystem services.

The species spanned a variety of functional groups (Figure 6). Primary producers dominated the species listed, while the other groups except for detritivores were almost equally represented. Furthermore, no single functional group was considered to represent a very high or high probability of threat (χ^2^ = 7.8, *df* = 5, *p* = 0.17).

### 3.4 Biogeographic regions under threat

The number of EU biogeographic regions under threat from the 66 species on the final list varied between thematic groups (GLM with quasi‐Poisson distribution; dispersion parameter = 0.44; analysis of deviance (type II): χ^2^ = 21.4, *df* = 4, *p *< 0.001), although the majority of the species were predicted to be of threat to two or more biogeographic regions (Table 1). A high number of the freshwater invertebrates and fish were anticipated to pose a threat to four or five biogeographic regions. In contrast, many of the marine species and vertebrates are likely to be restricted to two or three biogeographic regions. The terrestrial invertebrates and plant species are more evenly spread with more than two biogeographic regions predicted to be threatened in all cases. Two species were considered to pose a threat to five biogeographic regions, the Northern snakehead fish, *Channa argus*, and the black striped mussel, *Mytilopsis sallei*.

**Figure 7:**
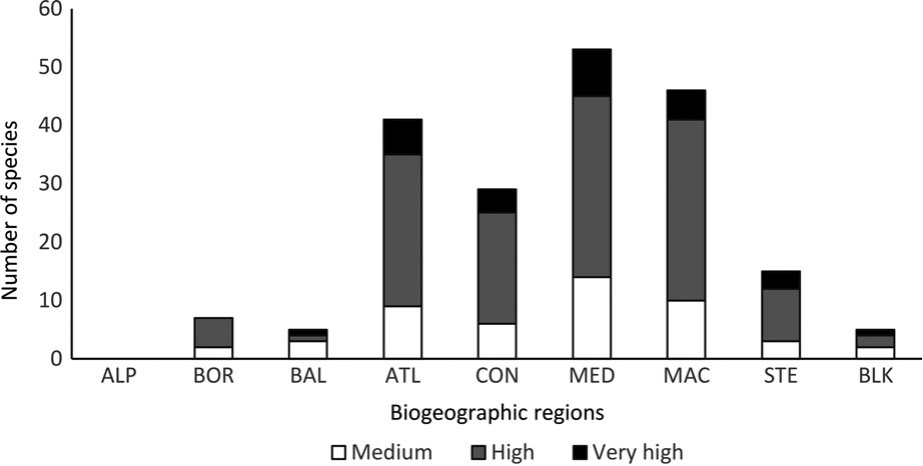
Number of species absent from Europe (n = 66) considered to have at least a medium probability of arrival, establishment, spread and impact on biodiversity and ecosystem services and the EU biogeographic regions they are predicted to establish within. Abbreviations: ALP = Alpine Region, BOR = Boreal Region, ATL = Atlantic Region, CON = Continental Region, MED = Mediterranean Region, MAC = Macaronesian Region, STE = Steppic Region, BLK = Black Sea Region

The Mediterranean, Continental, Macaronesian and Atlantic biogeographic regions are predicted to be the most threatened across all taxonomic groups (Figure 7; χ^2^ = 108.3, *df* = 7, *p *< 0.0001), whereas Baltic, Black Sea and Boreal biogeographic regions are predicted to be least at risk. The Alpine biogeographic region appears not to be under threat by any species. The terrestrial invertebrates, freshwater invertebrates and fish are likely to be of greatest threat to the Steppic biogeographic region.

## 4 DISCUSSION

Biological invasions involve complex processes and the ultimate success and impact of an alien species depends on many interacting biological, environmental and societal factors. The approach to horizon scanning proposed here attempts to prioritize potential future alien species in the EU acknowledging this complexity and the lack of evidence for many species under consideration. It is important to note the inherent biases in engaging experts through consensus methods (Sutherland & Burgman, 2015). However, employing techniques such as combining independent opinions and documenting the best available evidence can improve the reliability of judgements (Sutherland & Burgman, 2015). We not only captured independent scores as a first step in compiling the species list but we embedded the consensus methods within a framework that included literature review and impact assessment ensuring an evidence‐based approach which has applicability globally at various spatial and temporal scales. Ultimately our overarching aim was to systematically identify species considered to have a probability of arrival, establishment, spread and high impact on biodiversity and ecosystem services within the EU.

We identified 66 species that are currently absent from the EU and were considered to represent a medium, high or very high risk. The species identified in this horizon scanning exercise span a range of functional groups, with primary producers being numerically dominant. Escape from confinement is the pathway considered to be the most likely route of introduction for many species, particularly among plants and vertebrates. Both these patterns are consistent with already established aliens (DAISIE, 2009) and not surprising since many of the alien plants are anticipated to arrive as escapes from horticulture (Saul et al., 2017). Pathways within the stowaway categories are considered likely to increase in importance in terms of species introductions compared to the past (Chapman et al., 2016; Zieritz et al., 2017); this is particularly the case for marine species, but also for terrestrial invertebrates, and highlights the importance of increasing surveillance of transport vectors (Hulme, 2015; Saul et al., 2017; Pergl et al., 2017) and implementation of preventative measures. For example, the highly invasive fire ant, *Solenopsis invicta*, is likely to arrive as a stowaway in packaging (Inoue & Goka, 2009). It is important to consider the spread of IAS from countries adjacent to the region of interest but for the EU future major donor regions of IAS are also likely to be from further afield with introductions from Asia and the Americas anticipated to increase (Seebens et al., 2015; Zieritz et al., 2017). Thus, the pathways and origins of expected future IAS are similar to the major pathways of historic invasions in Europe (DAISIE, 2009).

Apart from some general patterns, alien species introduction events have a strong stochastic component. Therefore, it is important to recognize the imperfect nature of horizon scanning lists (Nehring, Kowarik, Rabitsch, & Essl, 2013). There are undoubtedly many species that have not been considered through this horizon scanning approach that could arrive in the future. However, involving a large number of people through a semi‐structured process to horizon scanning can inform the three‐stage hierarchical approach proposed by the CBD for managing the impacts of IAS. Communication and cross‐boundary collaborations extending beyond the EU, ensuring knowledge on IAS is shared between countries, are essential to ensure successful implementation of an IAS strategy (Hulme, Pyšek, Nentwig, & Vilà, 2009).

The breadth of biogeographic regions that are considered under threat by the species identified through the horizon scanning is striking, but it is notable that the Atlantic, Mediterranean, Continental and Macaronesian biogeographic regions are most at risk under current climate conditions, while the Alpine region is not. The Mediterranean biogeographic region is at risk because of the predicted arrival of Lessepsian potential IAS from the Indo‐Pacific exacerbated by the latest enlargement of the Suez Canal (Galil et al., 2015).

Climate warming is likely to play an important role in the future with respect to interactions with IAS, but not within the designated timeframe of 10 years (Walther et al., 2009; Cheng, Sakai, Matsushima, Yagi, & Hasegawa, 2010; Bellard et al., 2013). Some of the species that have been recorded but have not yet established might be able to reproduce and spread in future climates. This includes currently inhospitable regions, e.g. in the Alpine or Boreal region (Walther et al., 2009). It is essential that consideration is given to interactions between major drivers of change such as climate change, habitat destruction and pollution when predicting the likely establishment, spread and impacts of potential IAS.

The proposed lists provide a basis for prioritizing full risk assessments in order to comprehensively evaluate the threat posed by these species to the EU biodiversity. Completion of risk assessments for each species categorized as high or very high risk should be prioritized to validate the list and ensure that evidence of impacts is assessed in a rigorous and robust way. However, it would also be useful to assess a sample of those with medium risk scores as a way of checking the selection and ranking of species. Consideration of so many species requires a rapid method of assessment for arrival, establishment, spread and impact that enables effective, although approximate ranking. The crude bracketing of species as posing very high, high and medium threat was an effective way of managing the complexities of prioritizing such a long list of species spanning diverse taxonomic groups and environments. The experts were unanimously agreed that this approach increased their confidence in reaching a decision and reduced bias in the ranking, but note that the categorization is subjective. It is also important to remember that the scoring is to enable species to be prioritized for future formal risk assessment and that scores underpinned by detailed evidence should be collated during such risk assessment. Furthermore, we recommend conducting regular reviews of both the species rankings and future potential IAS that could threaten the EU, as demanded by the EU Regulation. For this purpose, dedicated species accounts should be considered and kept updated in the species data repository formally endorsed by the EU Regulation, i.e. EASIN (https://easin.jrc.ec.europa.eu/).

The focus of this horizon scanning exercise was only on the negative impacts of potential IAS on biodiversity and ecosystems, with some consideration on ecosystem service impacts. Systematic consideration of ecosystem services could form an integral part of a future horizon scanning exercise (Hulme & Vilà, 2017), and potentially evaluation of services and disservices. However, currently there is a lack of information to allow for a detailed and/or scientifically well‐informed assessment of ecosystem services including socio‐economic impacts, affecting the overall robustness of the scoring exercise (Roy, 2017). Therefore, biodiversity and ecosystem services impacts are recommended to be the core focus of a horizon scanning exercise with socio‐economic factors included where information is available. Additionally, improving the evidence‐base and developing frameworks for assessing socio‐economic impacts should be a priority.

Thematic groups ranked a similar number of species as very high, high or medium priority for risk assessment, with the exception of the terrestrial invertebrate group which listed fewer species than the others. For most terrestrial invertebrates, research on impacts is focused on productive sectors, such as forestry and agriculture, or human health and well‐being, rather than impacts on biodiversity. Substantial knowledge gaps for marine species (Ojaveer et al., 2015) and terrestrial invertebrates (Kenis et al., 2009; Nentwig & Vaes‐Petignat, 2014) have also been recognized. Indeed, all thematic groups struggled with a lack of information to some extent. For example, over all European alien species, impacts are reported for only 10% (Vilà et al., 2010), the main shortfall being poor understanding and documentation of impacts on ecosystem services (Roy, Schonrogge et al., 2014), although it is also recognized that a high proportion of alien species might not cause notable impacts (Roy, Preston et al., 2014) and the impacts of those that do are highly‐context dependent. Lack of information does not equate to absence of threat but a deliberately conservative approach was adopted whereby only those species with some supporting evidence of impacts on biodiversity were included in the list (Hulme et al., 2013). However, one of the advantages of using expert‐elicitation within a consensus approach to horizon scanning is the breadth of information sources drawn upon by the group members. Furthermore, evidence is accruing and new methods are ensuring robust and repeatable approaches for assessing environmental (Blackburn et al., 2014) and socio‐economic impacts (Bacher et al., 2018) including effects of IAS on ecosystem services.

Information provided by horizon scanning exercises is essential to support decision making on IAS, and to ensure an optimal use of the resources invested in prevention and early detection of possible invaders; activities that can require substantial economic investments. Therefore, regular review and refinement of the lists derived from such an approach will be critical. The horizon scanning method presented here could be extended in various ways particularly through inclusion of additional information on socio‐economic impacts (Bacher et al., 2018) but also identification and prioritization of emerging and promising IAS management methods, technologies or control actions (Shine et al., 2010; Ricciardi et al., 2017). Moreover, an important future priority is the management of arrival pathways of potential IAS considered to pose a major threat to biodiversity and ecosystem services (Essl et al., 2015; Vilà & Hulme, 2017) and this horizon scanning approach could inform pathway action plans.

## AUTHORS’ CONTRIBUTIONS

HER conceived the approach and led the manuscript. SB led the analyses. SB, FE, PG, SLR, TA, WR and RS contributed substantially to the writing of the manuscript. All authors contributed to the prioritization exercise including compilation of lists and rapid impact assessments and commented on the writing of the manuscript.
