# Supplementary material for: Developing a list of invasive alien species likely to threaten biodiversity and ecosystems in the European Union
Source: Glob Chang Biol. 2018 Dec 12;25(3):1032–48. doi: 10.1111/gcb.14527 (PMC7380041; doi:10.1111/gcb.14527)
Supplement: Supplementary file 1 [file GCB-25-1032-s001.docx]

**Developing a list of invasive alien species likely to threaten biodiversity and ecosystems in the European Union**

**Supporting Information**

**Supporting Information 1: Overview of approaches horizon-scanning methods**

Approaches to horizon scanning

Horizon scanning has historically included extensive literature reviews, to ascertain species of concern, and generally (but not always) some form of risk assessment. However, the importance of risk assessment tools is increasingly recognised as a component of approaches to identify potential future IAS not already present within a region ([Essl et al. 2011](#_ENREF_33)). Risk assessment tools based on a specified set of criteria have been developed for a number of countries. Many of these are used to prioritise alien species already present according to their impact ([Randall et al. 2008](#_ENREF_65)) although their potential for identifying future IAS that are not already present is recognised ([Roy et al. 2014a](#_ENREF_71)).

Strategic foresight is broadly defined as ‘the creative reorganization of information into future-oriented knowledge in the context of accelerated change and genuine uncertainty in high-velocity environments’ ([Copp et al. 2005](#_ENREF_23)) or simply a structured process for exploring alternative future states ([Copp et al. 2009](#_ENREF_25)). The different strategic foresight methods for conservation issues including (horizon) scanning, scenario planning and backcasting have been reviewed ([Crosti et al. 2010](#_ENREF_26)) and each have advantages and disadvantages, depending on the purpose of the exercise, data availability and data (un)certainty, and involvement of experts from different scientific, governmental or public domains. A number of structured approaches have been used for horizon scanning across a range of environmental disciplines (Table S1.1). Sutherland and Woodroof (2009) recognised that horizon scanning can be divided into six stages: (i) scoping the issue; (ii) gathering information; (iii) spotting signals; (iv) watching trends; (v) making sense of the future; and (vi) agreeing the response. However, within the context of horizon scanning to derive a list of IAS for prioritisation for risk assessment, (i) scoping the issue and (ii) gathering information are specifically pertinent.

Scoping the issue and gathering information are critical to all horizon scanning; the scope or key question must be explicitly identified and clearly understood by all involved in the horizon scanning. Ensuring that all participants understand the scope can require several iterations ([Sutherland, Woodroof 2009](#_ENREF_92)) and can be achieved through formal and structured interviews (Table S1.1). Gathering information can be achieved through a variety of approaches including open fora, questionnaires, literature review, modelling approaches, survey and experiment and expert workshops (Table S1.1) to supplement the prior knowledge of participants ([Sutherland, Woodroof 2009](#_ENREF_92)). Expert workshops including consensus approaches (modification of the Delphi technique, inclusive, transparent, and structured communication process, developed for systematic forecasting) have been extensively employed as an approach to horizon scanning within environmental science ([Sutherland et al. 2012c](#_ENREF_80); [Sutherland et al. 2014a](#_ENREF_81); [Sutherland et al. 2011a](#_ENREF_83); [Sutherland et al. 2013a](#_ENREF_84)).

**Table S1.1 Overview of broad approaches to horizon scanning including description, strengths and weaknesses. Examples relate to publications from the IAS-research area. Modified from Sutherland & Woodroof (2009).**

| Method | Detail | Strength | Weakness | IAS relevant examples |
| --- | --- | --- | --- | --- |
| Interview | One-to-one questioning; structured without debate or open | Good at getting key individuals perspectives on the future | No interaction between participants; possible bias due to selection of experts | – |
| Open for a | Online platform (Wiki) | Wisdom of the crowd, broadest possible range of contributors | Unstructured without quality control | – |
| Questionnaire | Expert consultation through pre-defined questions | Provides an overview of opinion on a specific theme | No interaction; possible bias due to selection of experts and how questions are phrased | – |
| Literature review | Extensive review of existing literature | Broad approach underpinned by existing knowledge (if peer-reviewed) | Unavailability of published reports or expert opinion; delay between observation and publication | (Parrott et al. 2009; Thomas 2011) |
| Modelling approach | Quantitative approach to derive predictions | Available data used to construct models to derive predictions | Depends on detailed life-history datasets which for many species are lacking | (Gallardo, Aldridge 2013) |
| Survey and experiment | Surveys of the environment in some cases coupled with experimentation | Realistic data derived | Labour intensive and expensive | (Richardson, Pyšek 2006) |

Inventory of existing horizon scanning methods

The Web of Science was used to derive horizon scanning methods relevant for assessment and critical review. A search for the keyword “horizon scanning” within the Web of Science revealed more than 1000 hits for the years 2000-2015 with some 200 publications from the Social Sciences and more than 900 from the “Science Technology” domain (including double-counting). A further refinement within the latter revealed 156 hits in the Research Area “Environmental Sciences Ecology” and 134 hits for “Agriculture”, although these include publications not related to the method, but to other contexts (e.g. soil horizons). We further refined our analysis to the Research Area “Biodiversity Conservation”, which delivered 27 hits, of which 20 were considered relevant after reading the abstracts of the papers (Table S1.2). The same search for the years 1990-1999 did not deliver a single relevant publication.

The scope of the horizon scanning examples listed in Table S1.2 was broad within the theme of biodiversity conservation and mainly in relation to identifying and prioritising issues rather than species. Only one of the examples is specific to IAS ([Caffrey et al. 2014](#_ENREF_15)) but again this exercise involved a prioritization approach used to elucidate the top 20 IAS issues in Europe. Most of the examples involved workshops in which experts were invited to participate, in many cases using consensus methods ([Sutherland et al. 2012a](#_ENREF_78)).

**Table S1.2 Publications on Horizon scanning derived from Web of Science within the Research Area “Biodiversity Conservation” (2000-2015).**

| Title | Scope | Method | Reference |
| --- | --- | --- | --- |
| Future novel threats and opportunities facing UK biodiversity identified by horizon scanning | Identify future developments of biodiversity in the UK up to 2050 that had not been important in the recent past | Consultation process with 452 people and consensus workshop with 35 representatives from environmental policy, academia and journalism | ([Sutherland et al. 2008](#_ENREF_82)) |
| One hundred questions of importance to the conservation of global biological diversity | Identify scientific questions most relevant for conservation practice and policy | Consultation process with 761 people, e-mail voting to short-list questions and consensus workshop with 33 representatives from international organisations, members of the Society for Conservation Biology, and academia | ([Sutherland et al. 2009](#_ENREF_77)) |
| The need for environmental horizon scanning | Calling for routine horizon scanning to decide on which issues researchers or practitioners should focus | Opinion paper | ([Sutherland, Woodroof 2009](#_ENREF_92)) |
| A horizon scan of global conservation issues for 2010 | Identify issues that could affect conservation of biological diversity | Consultation process of collecting, scoring and short-listing issues, followed by consensus workshop with subsequent e-mail discussion and re-scoring | ([Sutherland et al. 2010](#_ENREF_88)) |
| Horizon scan of global conservation issues for 2011 | Identify issues that could affect conservation of biological diversity | Consultation process with at least 158 people of collecting, scoring and short-listing issues, followed by consensus workshop with subsequent e-mail discussion and re-scoring | ([Sutherland et al. 2011a](#_ENREF_83)) |
| Methods for collaboratively identifying research priorities and emerging issues in science and policy | Identify priority policy-relevant research questions in the UK, USA and CAN relating to global conservation | Review paper. Methods should be based on inclusivity, openness, democracy | ([Sutherland et al. 2011b](#_ENREF_90)) |
| A horizon scan of global conservation issues for 2012 | Identify issues that could affect conservation of biological diversity | Consultation process with at least 253 people of collecting, scoring and short-listing issues, followed by consensus workshop with 22 participants | ([Sutherland et al. 2012c](#_ENREF_80)) |
| Making predictive ecology more relevant to policy makers and practitioners | Improve the capacity of testable predictions to aid policy makers and practitioners | Conceptual paper on different methods in predictive ecology | ([Sutherland, Freckleton 2012](#_ENREF_91)) |
| Enhancing the value of horizon scanning through collaborative review | Develop a process to identify appropriate responses by policy makers and practitioners | 12 environmental conservation organisations assessed collaboratively previously identified issues for their impact upon their organisations | ([Sutherland et al. 2012a](#_ENREF_78)) |
| What's on the horizon for macroecology? | Identify future challenges for the scientific field ‘macroecology’ (the analysis of large-scale, multi-species ecological patterns and processes) | Case-studies and literature analysis by the authors | ([Beck et al. 2012](#_ENREF_6)) |
| A horizon scanning assessment of current and potential future threats to migratory shorebirds | Examining future conservation issues of migratory shorebirds | E-Mail consultation process of scientists without scoring | ([Sutherland et al. 2012b](#_ENREF_79)) |
| A horizon scan of global conservation issues for 2013 | Identify issues that could affect conservation of biological diversity | Consultation process with at least 190 people of collecting, scoring and short-listing issues, followed by consensus workshop | ([Sutherland et al. 2013a](#_ENREF_84)) |
| A horizon scan of global conservation issues for 2014 | Identify issues that could affect conservation of biological diversity | Consultation process with at least 369 people of collecting, scoring and short-listing issues, followed by consensus workshop | ([Sutherland et al. 2014a](#_ENREF_81)) |
| Horizon Scanning: a new method for environmental and biodiversity conservation | – | Opinion paper | ([Jiang 2014](#_ENREF_44)) |
| Tackling invasive alien species in Europe: the top 20 issues. | A horizon scanning and issue prioritization approach used to elucidate the Top 20 IAS issues (as opposed to species) in Europe. | In excess of 100 expert delegates in a workshop setting | ([Caffrey et al. 2014](#_ENREF_15)) |
| Strategic foresight: how planning for the unpredictable can improve environmental decision-making | Highlighting ways foresight could play in environmental decision making | Review paper | ([Cook et al. 2014](#_ENREF_20)) |
| Evolutionary rescue in a changing world | Identify where the field of evolutionary rescue might develop | Case-studies and literature analysis by the authors | ([Carlson et al. 2014](#_ENREF_17)) |
| A horizon scan for species conservation by zoos and aquariums | Identify the top ten emerging issues for species conservation for the world zoo and aquarium community | Consultation process with more than 100 experts from the conservation and the zoo and aquarium community, followed by a workshop to short-list the top ten priority issues with potential to impact upon threatened species by 2020 | ([Gusset et al. 2014](#_ENREF_39)) |
| Seventy-one important questions for the conservation of marine biodiversity | Identify important questions to conserve and manage marine resources | 2 workshops with participants from academia, industry, government, and NGOs | ([Parsons et al. 2014](#_ENREF_63)) |
| Horizon scanning for invasive alien species with the potential to threaten biodiversity in Great Britain | See below | See below | ([Roy et al. 2014a](#_ENREF_71)) |
| A horizon scan of global conservation issues for 2015 | Identify issues that could affect conservation of biological diversity | Consultation process with at least 270 people of collecting, scoring and short-listing issues, followed by consensus workshop | ([Sutherland et al. 2015](#_ENREF_89)) |

**Supporting Information 2: Thematic groups**

Thematic groups established for the horizon scanning approach. Each group was led by two experts (group leaders) and included a number of additional contributors. Invited experts are shown in italics. All other contributors were project team members. All group leaders attended the workshop. The contributors marked in bold contributed to the preliminary consultation and post workshop discussions but did not attend the workshop. Four additional project team members attended the workshop: Jodey Peyton and Steph Rorke from the Centre for Ecology & Hydrology assisted with facilitation and data management; Ana Nieto and Mariana Garcia from the IUCN led the organisation of the workshop alongside Helen Roy. Ana-Cristina Cardoso from the JRC attended as a contributory partner.

| Thematic Group | Group leaders | Contributors |
| --- | --- | --- |
| Plants | Etienne Branquart  Montse Vilà | Franz Essl  Jan Pergl  Oliver Pescott  Philip Hulme  Sonia Vanderhoeven |
| Vertebrates | Riccardo Scalera  Sven Bacher | Piero Genovesi  Carles Carboneras  Tim Adriaens  Wojciech Solarz |
| Marine species | John Bishop  Argyro Zenetos | Juliet Brodie  Elizabeth Cook  Marco Faasse  Francis Kerckhof  Dan Minchin  Christine Wood |
| Terrestrial invertebrates | Wolfgang Nentwig  Alan Stewart | Jorgen Eilenberg  Marc Kenis  Cristina Preda  Wolfgang Rabitsch  Alain Roques  Karsten Schönrogge  Helen Roy |
| Freshwater invertebrates and fishes | David Aldridge  Emili García-Berthou | Gordon Copp  Belinda Gallardo  Elena Tricarico  Gerard van der Velde |

**Supporting Information 3: Databases screened for potential IAS**

List of selected 43 alien species databases particularly suitable for the purpose of a European horizon scanning on invasive alien species that have not yet arrived to the European Union (EU). The attributes in this table relate to the six criteria used to select this core set of alien species databases: i. Taxonomic, geographic and environmental coverage of the EU to allow assessment of the status of species within the EU (Geographic, taxonomic, and environmental coverage), ii. Taxonomic, geographic and environmental coverage of areas outside the EU that might be the origin of IAS possibly becoming introduced into the EU (Geographic, taxonomic, and environmental coverage), iii. Number of species included in the database, iv. Amount and quality of information available per species (species fact sheets and pathway information), v. functionality of the database including latest update, vi. Complementarity among the databases regarding taxonomic, geographic, and environmental coverage (Geographic, taxonomic, and environmental coverage).

| ID | Horizon scan | Continent | Data-base name | Full name | Website | No of species in data-base | Geographic scale | Geographic coverage | Taxonomic coverage | Environmental coverage | Species fact sheets and pathway information | Function-ality and last update | References (Examples) |
| --- | --- | --- | --- | --- | --- | --- | --- | --- | --- | --- | --- | --- | --- |
| 1 | 1 - most suitable | Asia | APASD | Asia-Pacific Alien Species Database | http://www.niaes.affrc.go.jp/techdoc/apasd/ | 317 | regional | Asia-Pacific (Japan, Malaysia, Philippines, Taiwan, Thailand, Vietnam, mainland China) | All taxonomic groups (Plants, animals, viruses, bacteria, fungi) | freshwater, terrestrial | Species fact sheets: YES Pathway information: NO | Fully functional Last update: 2014 | n.a. |
| 2 | 1 - most suitable | European seas | AquaNIS | Aquatic non-indigenous species | http://www.corpi.ku.lt/databases/index.php/aquanis/ | 1390 | regional | European seas with capability of global coverage | All taxonomic groups (all multicellular and some single celled aquatic taxa) | marine (incl.brackish) | Species fact sheets: YES Pathway information: YES | Fully functional Last update: 2015 | Olenin, S., Narščius, A., Minchin, D., David, M., Galil, B., Gollasch, S., Marchini, A., Occhipinti-Ambrogi, A., Ojaveer, H., Zaiko, A. (2014). Making non-indigenous species information systems practical for management and useful for research: An aquatic perspective. Biological Conservation 173: 98-107. |
| 3 | 1 - most suitable | Global | CABI Compendium | CABI Invasive Species Compendium | <http://www.cabi.org/isc/> | 8957 | Global | global | All taxonomic groups (incl. bacteria, fungi, protozoa, viruses) | freshwater, marine, terrestrial | Species fact sheets: YES Pathway information: YES | Fully functional Last update: 2015 | Pasiecznik, N. (2004). Pathways for plant introduction. CABI, Wallingford, UK, |
| 4 | 1 - most suitable | Europe | DAISIE | Delivering Alien Invasive Species Inventories for Europe | [www.europe-aliens.org](http://www.europe-aliens.org/) | >15000 | regional | wider European area (up to 94 countries/regionsincluding all EU‐27 states and Norway) | All taxonomic groups | freshwater, marine, terrestrial | Species fact sheets: YES Pathway information: YES | Fully functional Last update: 2012 (ongoing) | DAISIE (ed.) (2008). The Handbook of Alien Species in Europe, Springer-Verlag. |
| 5 | 1 - most suitable | Global | DIAS | FAO Database on Introductions of Aquatic Species | <http://www.fao.org/fishery/dias/en> | 5612 | Global | global | Fish, crustaceans, molluscs | freshwater, marine | Species fact sheets: YES Pathway information: YES | Fully functional Last update: 2015 | Welcomme, R.L. (1988). International introductions of inland aquatic species. FAO Fisheries Technical Paper 294, Food and Agriculture Organisation of the United Nations, Rome, 318 pp. |
| 6 | 1 - most suitable | Europe | EASIN | European Alien Species Information Network | <http://easin.jrc.ec.europa.eu/> | 16339 | regional | Europe | All taxonomic groups (incl. bacteria, fungi, protozoa, viruses) | freshwater, marine, terrestrial | Species fact sheets: YES Pathway information: YES | Fully functional Last update: 2015 | Trombetti, M., Katsanevakis, S., Deriu, I. and A.C. Cardoso (2013). EASIN-Lit: a geo-database of published alien species records. Management of Biological Invasions 4(3): 261-264. |
| 7 | 1 - most suitable | Europe, Africa, Asia | EPPO | European and Mediterranean Plant Protection Organization | <https://www.eppo.int/> | 91 | regional | Europe, N-Africa, Central Asia | Plants | terrestrial | Species fact sheets: YES Pathway information: YES | Fully functional Last update: 2015 | EPPO Bulletin <https://www.eppo.int/PUBLICATIONS/bulletin/bulletin.htm> |
| 8 | 1 - most suitable | Europe | ESENIAS | East and South European Network for Invasive Alien Species | [http://www.esenias.org](http://www.esenias.org/) | n.a. (species lists and factsheets still under construction) | regional | South and Eastern Europe (Albania, Bosnia and Herzegovina, Bulgaria, Croatia, Greece, Hungary, Italy, Kosovo under UNSC Resolution 1244/99, FYR Macedonia, Montenegro, Serbia, Slovenia, Romania, Turkey) | All taxonomic groups | freshwater, marine, terrestrial | Species fact sheets: currently not available Pathway information: currently not available | Under development Last update: 2015 | Zenetos, A., Katsanevakis, S., Poursanidis, D., Crocetta, F., Damalas D., Apostolopoulos G., Gravili C., Vardala-Theodorou, E. and M. Malaquias (2011). Marine alien species in Greek Seas: Additions and amendments by 2010. Mediterranean Marine Science, 12, 1: 95-120. |
| 9 | 1 - most suitable | Europe | EUROPHYT | European Union Notification System for Plant Health Interceptions | <http://ec.europa.eu/food/plant/plant_health_biosafety/europhyt/interceptions_en.htm> | e.g. >500 in 2011 | regional | Europe | Focus on plant pest but also notes host plants | terrestrial | Species fact sheets: NO Pathway information: YES | Fully functional Last update: 2015 | Europhyt. (2011). European Union Notification System for Plant Health Interceptions. available from http://ec.europa.eu/food/plant/europhyt/index_en.htm |
| 10 | 1 - most suitable | Global | GISD | Global Invasive Species Database | <http://www.issg.org/database/welcome/> | 891 | Global | global | All taxonomic groups | freshwater, marine, terrestrial | Species fact sheets: YES Pathway information: YES | Fully functional Last update: n.a. (2015?) | Invasive Species Specialist Group ISSG (2015). The Global Invasive Species Database. Version 2015.1 <http://www.issg.org/database > Accessed at 26-May-2015 |
| 11 | 1 - most suitable | Global | Global Marine Invasive Species Assessment | Global Marine Invasive Species Assessment | <https://www.conservationgateway.org/ConservationPractices/Marine/Pages/marineinvasives.aspx> | 330 | Global | global seas and oceans | All taxonomic groups | marine | Species fact sheets: YES Pathway information: YES | Fully functional Last update: 2015 (data until 2008) | Molnar, J.L., Gamboa, R.L., Revenga, C., and M.D. Spalding (2008). Assessing the global threat of invasive species to marine biodiversity. Frontiers in Ecology and the Environment 6(9), 485-492. |
| 12 | 1 - most suitable | South America, Central America | IABIN-I3N | Inter American Biodiversity Information Network (IABIN) - Invasive Species Network (I3N) | http://www.institutohorus.org.br/iabin/i3n/index.html | 436 (currently getting built up; for some countries functional – for others not yet) | regional | "Latin America" (Argentina, Bolivia, Brazil, Colombia, Chile, Costa Rica, Guatemala, Jamaica, Paraguay, Uruguay) | All taxonomic groups | freshwater, terrestrial | Species fact sheets: YES Pathway information: NO | For some countires functional; for most countires under development Last update: 2015 | n.a. |
| 13 | 1 - most suitable | Europe | ISEFOR | Increasing Sustainability of European Forests | [www.isefor.com](http://www.isefor.com/) | 996 | regional | Europe | Forest tree pests and pathogens (fungi, oomycetes and bacteria) | terrestrial | Species fact sheets: NO Pathway information: YES (focus on pathways, but no species specific pathway information readily available in a database or factsheets) | Fully functional (but no databases /factsheets) Last update: 2013 | Vannini, M., Franceschini, S. and A.M. Vettraino (2012). Manufactured wood trade to Europe: a potential uninspected carrier of alien fungi. Biological Invasions 14: 1991-1997. |
| 14 | 1 - most suitable | Central America | Malezas de Mexico | Weeds of Mexico / Malezas de Mexico | <http://www.malezasdemexico.net/> | appr. 1100 | national | Mexico | Plants (focus on “weeds”, but not all are alien) | terrestrial | Species fact sheets: YES Pathway information: NO | Fully functional Last update: n.a. | n.a. |
| 15 | 1 - most suitable | North America | NANIAD - Bugguide | Bugguide - List of non-native arthropods in North America | http://bugguide.net/node/view/32329 | 2273 | regional | North America | Arthropods | freshwater, terrestrial | Species fact sheets: YES Pathway information: NO | Fully functional Last update: 2015 | n.a. |
| 16 | 1 - most suitable | North America | NAS Database | Nonindigenous Aquatic Species Database (USGS) | <http://nas.er.usgs.gov/> | 1100 | national | USA | Invertebrates and vertebrates | freshwater, marine | Species fact sheets: YES Pathway information: YES | Fully functional Last update: 2015 | Several original sources in each fact sheet |
| 17 | 1 - most suitable | Europe | NOBANIS | North European and Baltic Network on Invasive Alien Species | <http://www.nobanis.org/> | 8739 | regional | 20 countries in Northern and Central Europe: Austria, Belarus, Belgium, Czech Republic, Denmark, Estonia, Finland, Faroe Islands, Germany, Greenland, Iceland, Ireland, Latvia, Lithuania, the Netherlands, Norway, Poland, European part of Russia, Slovakia, Sweden | All taxonomic groups | freshwater, marine, terrestrial | Species fact sheets: YES Pathway information: YES | Fully functional Last update: 2015 | Secretariat of NOBANIS (2012): Risk­mapping for 100 non­native species in Europe. Copenhagen. <http://www.nobanis.org/files/Riskmapping_report.pdf> |
| 18 | 1 - most suitable | Europe | Q-bank | Q‐bank – Comprehensive Databases on Regulated Plant Pests | <http://www.q-bank.eu/> | appr. 2000 | regional | Partners from 20 countries including The Netherlands, Belgium, United Kingdom, France, Denmark and Italy | Fungi, arthropods, plants, nematodes, viruses, phytoplasmas | terrestrial | Species fact sheets: YES Pathway information: YES | Fully functional Last update: 2015 | Bonants, P., Edema, M. and V. Robert (2013). Q‐bank, a database with information for identification of plant quarantine plant pest and diseases. EPPO Bulletin 43.2: 211-215 |
| 19 | 1 - most suitable | Africa | WIP | Weeds and Invasive Plants (South Africa) | <http://www.agis.agric.za/wip/> | appr. 600 | national | South Africa | Plants | freshwater, terrestrial | Species fact sheets: YES Pathway information: NO | Partly not functional Last update: n.a. (probably not very often actualized) | Henderson, L. and C.J. Cilliers (2002). Invasive aquatic plants. Plant Protection Research Institute Handbook No. 16, Agricultural Research Council, Pretoria. |
| 20 | 1 - most suitable | North America | [www.invasive.org](http://www.invasive.org/) | The Bugwood Network (University of Georgia) | [http://www.bugwood.org/ www.invasive.org](http://www.bugwood.org/%20%20%20%20%20%20%20%20www.invasive.org) | 2908 | national | USA | All taxonomic groups | freshwater, marine, terrestrial | Species fact sheets: YES Pathway information: NO | Fully functional Last update: 2014 | n.a. |
| 21 | 2 - suitable | Global | GCW | Global Compendium of Weeds | <http://www.hear.org/gcw/scientificnames/scinameo.htm> | >28000 | global | global | Plants (Weeds) | freshwater, marine, terrestrial | Species fact sheets: YES (but poor) Pathway information: NO | Fully functional Last update: 2007 | Randall, R.P. (2002). A global compendium of weeds. Second Edition, Publisher: Department of Agriculture and Food, Western Australia. |
| 22 | 2 - suitable | Australia (and Oceania) | HEAR/PIER | Invasive species information for Hawaii and the Pacific | http://www.hear.org/ | n.a. | regional | Pacific Islands | Plants | freshwater, marine, terrestrial | Species fact sheets: YES (but poor) Pathway information: NO | Limited functionality, may close soon Last update: 2012 | US Forest Service, Pacific Island Ecosystems at Risk (PIER). Online resource at http://www.hear.org/pier/ accessed 26-May-2015 |
| 23 | 2 - suitable | Global | IBIS | Island Biodiversity and Invasive Species Database | [http://ibis.fos.auckland.ac.nz](http://ibis.fos.auckland.ac.nz/) | n.a. | global | global islands | All taxonomic groups | freshwater, marine, terrestrial | Species fact sheets: YES Pathway information: NO | Fully functional Last update: 2015 | Invasive Species Specialist Group –ISSG (2012). Island Biodiversity and Invasive Species Database -IBIS Version 2012.1 <http://ibis.fos.auckland.ac.nz/ > |
| 24 | 2 - suitable | Australia (and Oceania) | Invasive Invertebrate Threats | Invasive Invertebrates in Natural Ecosystems (New Zealand) | <http://www.landcareresearch.co.nz/research/biocons/invertebrates/> | appr. 60 | national | New Zealand | Invertebrates | freshwater, terrestrial | Species fact sheets: YES Pathway information: YES | Fully functional Last update: 2015 | n.a. |
| 25 | 2 - suitable | North America | Invasive Species Encyclopedia | Invasive Species in Canada (Wildlife Federation Canada) | http://cwf-fcf.org/en/discover-wildlife/resources/encyclopedias/invasive-species/ | 414 | national | Canada | all taxonomic groups | freshwater, marine, terrestrial | Species fact sheets: YES Pathway information: YES | Fully functional Last update: 2015 | n.a. |
| 26 | 2 - suitable | North America | NEMESIS | National Exotic Marine & Estuarine Species Information System (SERC) | http://invasions.si.edu/nemesis/databases.html | 137 | national | USA | Invertebrates | marine | Species fact sheets: YES Pathway information: YES | Currently geeting restricted, but seemingly fully functional Last update: n.a. (2015?) | Fofonoff, P.W., Ruiz, G.M., Steves, B. and J.T. Carlton (2014). National Exotic Marine and Estuarine Species Information System. http://invasions.si.edu/nemesis/. Access Date: 26-May-2015 |
| 27 | 2 - suitable | Australia (and Oceania) | NIMPIS | National Introduced Marine Pests Information System | http://data.daff.gov.au/marinepests/#srchByNameOrNumber | >100 | national | Australia | all taxonomic groups | marine | Species fact sheets: YES Pathway information: YES | Fully functional Last update: n.a. (2015?) | NIMPIS (National Introduced Marine Pest Information System). (2009). Web publication <http://www.marinepests.gov.au/nimpis>. Date of access: 26-May-2015 |
| 28 | 2 - suitable | North America | Pest Tracker | PestTracker (NAPIS Purdue University; USDA-APHIS) | <http://pest.ceris.purdue.edu/pests.php> | 617 | national | USA | All taxonomic groups (plants, animals, fungi, bacteria, viruses) | terrestrial | Species fact sheets: YES Pathway information: NO | Fully functional Last update: 2015 | n.a. |
| 29 | 2 - suitable | North America | USDA APHIS Regulated Pest List | USDA APHIS Regulated Pest List (www.invasive.org) | <http://www.invasive.org/species/list.cfm?id=4> | 239 | national | USA | All taxonomic groups (plants, animals, fungi, bacteria, viruses) | terrestrial | Species fact sheets: YES (but rather poor) Pathway information: NO | Fully functional Last update: 2009 | n.a. |
| 30 | 2 - suitable | North America | USDA-PLANTS | Federal and State Noxious Weeds (USDA-PLANTS) | <http://plants.usda.gov/java/noxComposite> | 679 | national | USA | Plants | freshwater, terrestrial | Species fact sheets: YES Pathway information: NO | Fully functional Last update: n.a. (2014?) | n.a. |
| 31 | 2 - suitable | Australia (and Oceania) | Weeds Australia database | Weeds Australia database | <http://search.weeds.org.au/> | 481 | national | Australia | Plants | freshwater, terrestrial | Species fact sheets: NO Pathway information: NO | Fully functional Last update: n.a. | Thorp, J.R., Wilson, M.W. (1998 onwards) Weeds Australia - [www.weeds.org.au](http://www.weeds.org.au) Date of access: 26-May-2015 |
| 32 | 3 - possibly suitable | North America | AKEPIC | Alaska Exotic Plant Mapping Project (Alaska) | <http://aknhp.uaa.alaska.edu/botany/akepic/> | 160 | (sub-)national | USA | Plants | freshwater, terrestrial | Species fact sheets: YES Pathway information: partly | Fully functional Last update: 2015 | [AKEPIC (Year). Alaska Exotic Plant Information Clearinghouse database (http://aknhp.uaa.alaska.edu/maps/akepic/). Alaska Natural Heritage Program, University of Alaska, Anchorage. Date of access: 26-May-2015](http://aknhp.uaa.alaska.edu/maps/akepic/) |
| 33 | 3 - possibly suitable | Europe | Artsdatabanken | Artsdatabanken | http://www.artsdatabanken.no/fremmedearter | 2595 | national | Norway | All taxonomic groups | freshwater, marine, terrestrial | Species fact sheets: YES Pathway information: YES | Fully functional Last update: n.a. (2015?) | Gederaas, L., Moen, T.L., Skjelseth, S. and L.-K. Larsen (eds.). Alien species in Norway– with the Norwegian Black List 2012. The Norwegian Biodiversity Information Centre, Norway. |
| 34 | 3 - possibly suitable | Global | Avibase | Avibase – the world bird database | <http://avibase.bsc-eoc.org/checklist.jsp?lang=EN> | 10000 (but most are not IAS!!) | global | global | birds | freshwater, marine, terrestrial | Species fact sheets: YES Pathway information: NO | Fully functional Last update: 2015 | McKinney, M.L. (2006). Correlated non-native species richness of birds, mammals, herptiles and plants: scale effects of area, human population and native plants. Biological Invasions 8: 415-425. |
| 35 | 3 - possibly suitable | Europe | Especies introducidas en Canarias | Especies introducidas en Canarias | http://www.interreg-bionatura.com/especies/ | appr. 1000 | (sub-)national | Canary Islands (Spain) | animals, plants, fungi, algae | freshwater, marine, terrestrial | Species fact sheets: YES Pathway information: NO | Fully functional Last update: 2014 | Arechavaleta, M., Rodríguez S., Zurita N. & A. García (Coord.) (2010). Lista de especies silvestres de Canarias (hongos, plantas y animales terrestres) 2009. Gobierno de Canarias. 579 pp. |
| 36 | 3 - possibly suitable | Global | FishBase | FishBase – A Global Information System on Fishes | [http://www.fishbase.org](http://www.fishbase.org/) | 32900 (but most are not IAS!!) | global | global | fish | freshwater, marine | Species fact sheets: YES Pathway information: NO | Fully functional Last update: 2015 | Froese, R. and D. Pauly (eds.) (2014). FishBase. World Wide Web electronic publication. www.fishbase.org, version (05/2015). |
| 37 | 3 - possibly suitable | Europe | Flora of Iceland | Flora of Iceland | <http://www.floraislands.is/index.html> | 5610 (but most are not IAS!!) | national | Iceland | Plants (incl. mosses), Lichens, Fungi, Algae | freshwater, terrestrial | Species fact sheets: YES (only in Icelandic Pathway information: YES | Fully functional Last update: n.a. | n.a. |
| 38 | 3 – possibly suitable | Global | GBIF | Global Bio-diversity Informa-tion Facility | http://www.gbif.org/ | appr. 1 600 000 (but most are not IAS!!) | global | global | All taxonomic groups (animalia, archaea, bacteria, chromista, fungi, incertae, plantae, protozoa and viruses) | Fresh-water, marine, terrest-rial | Species fact sheets: YES Pathway information: YES | Fully functional Last update: 2015 | Berendsohn, W.G., Vishwas C. and J. Macklin (2010). Summary of Recommendations of the GBIF Task Group on the Global Strategy and Action Plan for the Digitisation of Natural History Collections. Biodiversity Informatics 7.2. |
| 39 | 3 – possibly suitable | Global | GPDD | Global Pest and Disease Database (USDA / PPQ) (restricted access) | <https://www.gpdd.info/> | 3700 | Global | Global | n.a. | freshwater, marine, terrestrial | Species fact sheets: n.a. Pathway information: n.a. | Restricted access Last update: n.a. | n.a. |
| 40 | 3 – possibly suitable | North America | GRIN | Germplasm Resources Informa-tion Network (USDA) | http://www.ars-grin.gov/npgs/index.html | n.a. | National | USA | Plants, Animals, Microbes | freshwater, marine, terrestrial | Species fact sheets: NO Pathway information: NO | Fully functional Last update: 2010 | n.a. |
| 41 | 3 – possibly suitable | North America | Nature Serve | Nature Serve Explorer | http://www.natureserve.org/conservation-tools/data-maps-tools/natureserve-explorer | 70000 (but including ecosystems and native species!!) | Regional | USA & Canada | Plants, Animals, Fungi | freshwater, marine, terrestrial | Species fact sheets: n.a. Pathway information: NO | Much information but not fully functional. Most relevant tool (i.e. “Nature Serve Explorer”) was not functional at last check (6.7.2015) Last update: 2015. | n.a. |
| 42 | 3 – possibly suitable | North America | NBIC | National Ballast Water Informa-tion Clearing-house (SERC) | <http://invasions.si.edu/nbic/> | n.a. | national | USA | n.a. | marine | Species fact sheets: NO Pathway information: NO | Fully functional Last update: 2015 | [National Ballast Information Clearinghouse (2014). NBIC Online Database. Electronic publication, Smithsonian Environmental Research Center & United States Coast Guard. Available fromhttp://invasions.si.edu/nbic/search.html; searched 26-May-2015](http://invasions.si.edu/nbic/search.html) |
| 43 | 3 – possibly suitable | North America | NISIC | National Invasive Species Informa-tion Center (USDA) | http://www.invasivespeciesinfo.gov/about.shtml | 150 | national | USA | Plants, Animals, Microbes | Fresh-water, marine, terrest-rial | Species fact sheets: YES Pathway information: YES | Fully functional Last update: 2015 | n.a. |

**Supporting Information 4:** **Specific approaches adopted by each thematic group to the compilation of preliminary horizon scanning lists of IAS**

***Marine Group***

**Group leaders**: John Bishop and Argyro Zenetos

**Contributors**: Juliet Brodie, Elizabeth Cook, Marco Faasse, Francis Kerckhof, Dan Minchin, Christine Wood

A long list was created of alien species already introduced within European seas, but with limited distributions, detailing their occurrence in EU member states and adjacent non-EU countries in the Black Sea and eastern Mediterranean Sea. EASIN, DAISIE, AquaNIS and some recent literature sources were consulted. In parallel with this, a second list was made of species considered invasive in other world seas with environmental conditions similar to Europe, using databases (Supporting Information 2) and consultation of primary literature.

Care was taken to include species representative of the most important taxonomic groups globally amongst alien species. However, phytoplankton species were not considered because of lack of expertise within the group and persistent problems with ascertaining the status of species as alien or native.

Candidate species that scored reasonably highly but were removed from the list included:

1. Taxa presenting problems of identification as members of unresolved species complexes, or at least not considered reliably separable from their close relatives: *Asterias amurensis, Streblospio gynobranchiata, Phallusia nigra, Lithophyllum yessoense* and *Kappaphycus alvarezii*.
2. Species occurring in fewer than three member states, but judged too well established in the EU based on the criterion given during the workshop, “limited distribution in the EU of a few, small, isolated populations”: *Schizoporella japonica* (UK only, but several large populations), *Chama pacifica* (well established and spreading in Greece and Cyprus), *Chrysonephos lewisii* (Italy and France), *Celtodoryx ciocalyptoides* (large populations in the Netherlands and southern Brittany), *Oithona davisae* (pelagic, with populations in the southern North Sea, and invasive in the Black Sea), *Fenestrulina delicia* (a species present in the southern North Sea and English Channel, elsewhere off the UK, and abundant inshore off the Netherlands).

The following high-ranking species were included in the list on available information as being represented by either one or a small number of isolated small populations within a restricted region: *Pterois miles* (possibly established in Cyprus), *Penaeus azteca* (established in Greece) and *Homarus* *americanus* (possibly established locally in Sweden).

It is likely, given additional time to select and score marine species and to debate the scores, that the species ultimately selected, and their order in the list, would have changed somewhat. The scores on the preliminary list brought to the workshop were altered substantially during debate within the specialist group, which is appropriate, but the final scores represent a general classification and not a definitive statement.

While the principle of environmental matching was used in identifying species likely to become invasive in Europe, species native to sub-tropical and tropical regions may have the ability to adapt to and colonise cooler environments. Adaptation from cooler to much warmer environments is also documented, for instance the North Pacific starfish Asterias amurensis invasive in southern Australia. The ultimate ranges of such species cannot, therefore, always be predicted.

Deciding and maintaining the appropriate geographical and taxonomic balance of the species assessed is a challenge in exercises such as these, particularly in the European marine context given potential domination by the highly invaded Mediterranean biota and the marked biogeographical division between the Mediterranean Sea and Atlantic coasts.

Some pronounced changes in pathways and vectors bringing IAS to Europe are underway. The development of a second Suez Canal is expected to increase markedly the rate of arrival of Indo-Pacific species in the south-eastern Mediterranean. With the decline of Arctic ice cover, the expected increase in shipping traffic via northern routes between the Atlantic and Pacific is likely to result in many north-western Pacific species entering the North Atlantic, with impacts on northern European seas. The substantial present problems of European oyster culture caused by oyster herpesvirus infections may stimulate the importation of replacement stock from distant regions, with attendant hitch-hikers, potentially reproducing the influx of IAS associated with analogous crises in the French oyster industry in the 1970s. Continuing global warming and ocean acidification might also accelerate the rate of change of species’ distributions. Conversely, the imminent adoption of the international convention on ballast water management could substantially suppress the ballast water vector.

***Plant group***

**Group leaders**: Etienne Branquart and Montse Vila

**Contributors**: Franz Essl, Jan Pergl, Oliver Pescott, Philip Hulme, Sonia Vanderhoeven

The plant group adopted an approach based on invasion history elsewhere and climate suitability in Europe as the best predictors to identify potential IAS. The focus was mostly on horticulture as the major intentional pathway; however, potential species to be used as biofuel and macrophytes to be used as ornamental plants or that could be accidentally introduced were also explored. Ferns and mosses were included but not algae.

The following databases were used:

*Horticultural plants already introduced in Europe*

For horticultural plants, by far the most important single pathway of alien plants, we selected candidate species from a subset of species which are included in the European Garden Flora (i.e. all plants cultivated in Europe and not native to Europe), which are not yet present as established aliens in Europe, but have already established in other continents. This list was compiled for a recent publication on naturalization risks of alien garden plants (Dullinger et al. 2017).

For the Horizon Scanning Project, a standardized taxonomy to The Plant List (http://www.theplantlist.org/) was employed, and species were ranked by the number of regions / continents they currently invade outside Europe. This was done based on the recently completed global alien plant distribution database, the GloNAF database (https://glonaf.org/). This database is not open access and so was not included in Supplementary Information 2. However, GloNAF has been used in a number of recent publications ([Essl et al. 2015](#_ENREF_32); [Seebens et al. 2015](#_ENREF_74)). In total 290 species were identified as naturalized in at least three continents outside Europe, but not in Europe.

In a second step, Species Distribution Models for these species were made based on GBIF data etc. to evaluate which of these species are likely to establish currently and under future climates in Europe. This may provide some guidance to identify future likely invaders. Species were ranked according to the number of grid cells under moderate climate change (RCP 2.6 climate change scenario), but the sequence of species would also be similar for current climate. We screened the top 102 species for Horizon Scanning for documented impacts on biodiversity and ecosystem services based on available scientific publications and information systems (CABI ISC, IUCN, etc).

*Plant species selection from lists of IAS in USA and Japan*

Species known to be environmental weeds in USA or in Japan (http://www.nies.go.jp/biodiversity/invasive/index_en.html, accessed 15. April 2015) were extracted from local databases, from which were excluded:

1. Species native to EU countries, based on information from the DAISIE database, the CABI Invasive Species Compendium and Flora Europaea.
2. Species established in more than 5 EU countries, based on the same information sources (hereafter considered as widespread).

In a second step, the list of species was further refined in selecting only species with a good climate match with EU conditions (exclusion of tropical species) and with strong documented impacts on biodiversity and ecosystem services (environmental weeds) in CABI ISC, IUCN GISD, EPPO or NatureServe databases.

For USA, 222 species with a medium to high environmental impact (I-Rank) value were extracted from the NatureServe database (<http://www.natureserve.org/>, accessed April 2015). They were afterwards shared between the following categories:

1. Species native to EU countries: n = 105
2. Widespread alien species in Europe (established in more than 5 countries): n = 24
3. Absent or emergent species in Europe: n = 93

For Japan, 143 species were extracted from the list of invasive alien plants. They were afterwards shared between the following categories:

1. Species native to EU countries: n = 4
2. Widespread alien species in Europe (established in more than 5 countries): n = 39
3. Absent or emergent species in Europe: n = 57

*Plant species selection from lists of IAS in New Zealand*

The selection was based on:

1. Identification of all naturalized species known to invade protected areas in NZ (as a measure of potential ecological impact).
2. Selection on those species not native to Europe and not yet naturalized in Europe.
3. Focus on species that occur in at least two administrative regions in NZ, as a measure of potential spread.
4. Target the subset of these species that are recent introductions and naturalization e.g. from around 1950 onwards potentially highlighting shorter lag phases.
5. Cross reference with horticulture websites in Europe to see whether the species has already been introduced.

*Plant species selection from lists of IAS in Australia*

Screening of this database was partial, because the database of the introduced flora of Australia and its weed status (Randall 2007) includes information for > 25 000 species, whether or not naturalized over there. The list of 102 horticultural species was cross-checked with the invasiveness information of Randall (2007). Weedy status is collected from Australia and also from other areas in the world. A focus was put on environmental weed status and invasive status in Randall's typology. About 20 species came out of the 102 horticultural taxa.

*Plant species selection from lists of IAS in other Mediterranean Regions*

The database come from an Horizon Scanning conducted for Spain ([Andreu, Vilà 2010](#_ENREF_1)) that included more than 80 species known to be invasive in non-European Mediterranean regions, namely, N Africa, California, and Mediterranean-climate regions in Chile and Australia. For these species the Phelung WRA ([Pheloung et al. 1999](#_ENREF_64)) and the Weber WRA ([Weber, Gut 2004](#_ENREF_98)) for Central Europe had ranked them by their potential invasive status.

***Vertebrate group***

**Group leaders**: Riccardo Scalera and Sven Bacher

**Contributors**: Piero Genovesi, Carles Carboneras, Tim Adriaens, Wojciech Solarz

The selection of species was carried out in two successive stages. During the compilation of the preliminary list, species were selected with a limited range in Europe (but neither native to Europe, nor cryptogenic) or not yet present, and with high risk of being invasive in the EU, mostly taken from DAISIE/EASIN, plus some additions from a few reports (see Parrott et al. 2009). The information was cross-checked on GISD.

In general, the overall approach suggested by Roy et al. (2014), and Faulkner et al. (2014) was followed. We added a few species selected through expert opinion by Sven Bacher and Riccardo Scalera. In total, 51 species were selected (one of which was later removed because it is native to the EU).

Once the preliminary list was completed, the compilation of an additional list of species through two methodologies similar to the previous one was initiated:

1. using the revised GISD to try define a few additional vertebrate species to consider in the HS. Species causing impacts on endangered species (IUCN Red List CR, EN, VU) in other regions of the world, and not present in Europe were selected then the information within GISD was used to compile the required information.
2. a literature search of the DAISIE and GB NNSIP databases and the works of the SEO/BirdLife Working Group on Exotic Species, including the List of introduced birds in Spain and Europe by Santos Clavell & Sol (2007). Preference was given to the species that had been detected in the wild in Europe but were yet to establish self-sustaining populations. We added two species selected through expert opinion of W. Solarz. In total, 39 species were selected (two of which were later removed because they are native to the EU).

***Terrestrial invertebrate group***

**Group leaders**: Wolfgang Nentwig and Alan Stewart

**Contributors**: Karsten Schonrogge, Wolfgang Rabitsch, Marc Kenis, Cristina Preda, Helen Roy, Alain Roques, Jørgen Eilenberg

The members of the terrestrial invertebrate group had expertise across Insecta, Arachnida, Gastropoda, Annelida, Platyhelminthes, and Nematoda. Each group member was asked to submit lists, using the databases identified in Supporting Information 2 but also other sources, of potential IAS of EU concern which resulted in a combined list of 54 species. Fifteen species already present in the EU were placed into a separate list, leaving 39 species not yet present in the EU. All species were scored by the group members according to the guidance provided. Some group members refrained from scoring all species, because of a lack of expertise in some specific groups (e.g. spiders). Queries about the guidance were discussed within the group and if necessary passed to the project leader for clarification so that information would be passed to other groups as well.

A group score for each species was calculated as the mean over the scores of individual group members. Each of the lists was divided into species with high, medium and low scores, with boundaries set at 80 and 40. Setting the cut-off score at 80 produced a top 18 species, the largest groups being wood-boring beetles (9 species) and ants (5 species). The unified list was circulated to group members for consideration and as a basis for discussion at the workshop.

At the workshop, the following steps were taken:

1. All confidence / certainty scores were used in the discussions about individual species but were not used in any quantitative way.
2. Some species were reinstated to the main list because they are present only in a “few small isolated populations” such as *Arthurdendyus triangulatus* (NZ flatworm) only present in part of UK.
3. Species listed on EPPO lists (A1, A2) were included in the main list because they are not yet part of any EU regulation (following guidance from EC).
4. Four alien species of scolytid beetles attacking conifers were considered in the pre-list but were removed from the final list of the Horizon Scanning exercise because we took into account that the Annex II of the European Directive regarding plant health (2000/29/CE) mentions the regulation of all non-European scolytids as “*harmful organisms whose introduction into and spread within all member states should be banned*“. More specifically, Annex II indicates that the subject of contamination is “*the plants of conifers, over 3 m in height, other than cones and seeds, wood of conifers with bark and isolated bark of conifers, originating in non-European countries*”. Note that these four species associated with conifers pose a risk to the environment, not only to forestry. They consist of *Polygraphus proximus, the* Sakhalin-fir bark beetle, which has been introduced from the Far East into both Siberia and European Russia (Saint Petersburg, Moscow), and is a vector of pathogenic fungi killing fir trees. This insect is thus a threat to fir stands in Europe (Horizon scanning score 134). Three *Dendroctonus* species native to North America constitute also threats for native pine and spruce stands in Europe: namely *D. ponderosae*, the mountain pine beetle, score 95; *D. valens,* red turpentine bark beetle, score 93; and *D. rufipennis,* the spruce beetle, score 75.
5. Four species of ambrosia beetles were initially removed from the list, but this was later discovered to be due to an erroneous reading of Annex II of the Regulation which applies only to scolytids associated with conifers. Thus, these 4 species were re-instated to the terrestrial invertebrates list, but only AFTER the overall list had been finalised by consensus. One species, *Pityophthorus juglandis* (score = 133), a vector of pathogenic fungi killing walnut trees, would have been included in the top 100 of the overall list.
6. Three species were removed following the workshop because it was evident that these species were already included in the EU plant health legislation (amendments to Council Directive 2000/29/EC as of 30.06.2014): *Agrilus planipennis* (score 500, very high), *Dendrolimus sibirus* (score 128, medium), and *Agrilus anxius* (score 96, medium).
7. *Culex quinquefasciatus* was deleted (score < 80) because the freshwater group included it within their list.
8. The small hive beetle, *Aethina tumida,* was considered in the pre-list, but was removed from the final list of the Horizon Scanning exercise because we considered it is already regulated.

**Preliminary list of freshwater invertebrates**

Five primary databases and lists were scanned comprehensively to derive our long list:

| NOBANIS full list | <http://www.nobanis.org/> |
| --- | --- |
| DAISIE 100 Worst | <http://www.europe-aliens.org/speciesTheWorst.do> |
| GISD 100 Worst | http://www.issg.org/database/welcome/ |
| DIAS full list | <http://www.fao.org/fishery/dias/en> |
| ISE-CANADA full list | www.issg.org/database/ |

Species restricted to tropical climates (in both their native and invaded range) were removed from the list. While this deselection was questioned by some colleagues at the meeting in Brussels, we would stress that this selection was based on species with NO record in a bioclimatically suitable region, and as such would have no evidence base on which to consider them a threat in the next decade. Species that were associated solely with brackish waters were sent to the Marine Subgroup for screening. The team then identified additional species which do not appear on any lists but are considered to be an emergent threat. A summary of our methodology is given in Figure S1.


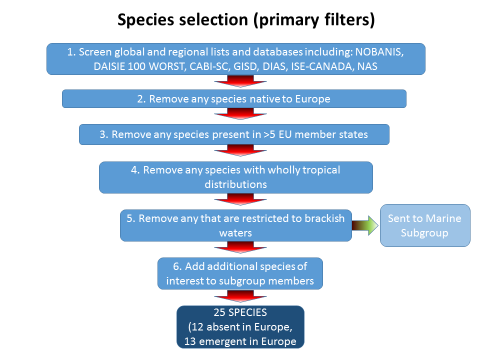


**Figure S1**. Summary of the process used by the terrestrial invertebrate group to select species for scoring.

*Scoring*

Scoring used a three-round Delphi process as recommended by Sutherland ([www.conservationevidence.com](http://www.conservationevidence.com)). Each species was scored blind by at least three experts from the subgroup (D. Aldridge, B. Gallardo, G. van der Velde, E. Tricarico). The project template was used and scorers were encouraged to provide an evidence base for their conclusions using the comments box and supporting references. Median scores for risk and confidence were calculated and circulated to all assessors. Collective A*B*C*D scores were generated by multiplying the median scores for each category. Collective confidence was scored by taking medians from the assessors for each box in the spreadsheet. There was broadly remarkable agreement in the scores given by each assessor. Where assessors disagreed with the collective median they were invited to challenge the score with a reasoned, evidence-based case. In each instance, the assessors were then asked to rescore that species. A summary of the scoring methodology is given in Figure S2.


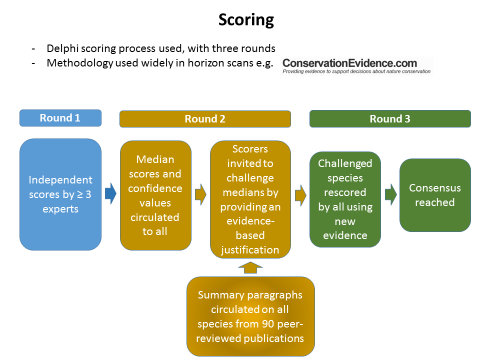


**Figure S2**. Summary of the three-round Delphi process used to reach consensus on species scoring and prioritisation.

Using this scoring the team identified our 12 most highly scoring species and these were entered into the collective ranking process.

**Supporting Information 5: Impact and confidence Scores**

Impact categories, based on the likely mechanisms of impact ([Blackburn et al., 2014](#_ENREF_1)), were circulated to the thematic groups for consideration during the preliminary scoring phase of the horizon scanning. Experts were referred to the ecosystem services framework described in “Organisation and running of a scientific workshop to complete selected invasive alien species (IAS) risk assessment ARES(2014)2425342 - 22/07/2014” ([Roy et al., 2014](#_ENREF_3)). The EU Habitats Directive was referred to for consideration of the colonisation of high conservation value habitats.

| **Impact category** | **Mechanisms** |
| --- | --- |
| **Adverse impact on native species** | 1. Competition 2. Predation 3. Hybridization 4. Disease transmission 5. Parasitism 6. Poisoning / toxicity 7. Bio-fouling 8. Grazing / herbivory / browsing 9. Interactions with other IAS |
| **Adverse impact on, or alteration of, ecosystem function** | 1. Modification to nutrient cycling 2. Physical modification of the habitat 3. Modification of natural succession 4. Disruption of food webs |
| **Adverse impacts on ecosystem services** |  |
| **Colonisation of high conservation value habitats** |  |

Descriptors of the five point impact scoring system circulated to the thematic groups for implementation during the preliminary scoring phase of the horizon scanning (Minimal concern =1; Minor =2; Moderate = 3; Major = 4; Massive = 5).

| **Target of impact** | **Impact score** | **Definition** |
| --- | --- | --- |
| **Impact on common species and habitats** | Minimal concern | Localised and moderate (or regional and minor) losses, easy to reverse |
|  | Minor | Regional and moderate losses, difficult to reverse |
|  | Moderate | Regional and major (or widespread and moderate) losses, difficult to reverse |
|  | Major | Widespread and major losses, irreversible |
|  | Massive | Not achievable for common species and habitats |
| **Impact on species and habitats of conservation importance** | Minimal concern | Localised and minor losses, easy to reverse |
|  | Minor | Localised and moderate (or regional minor) losses, difficult to reverse |
|  | Moderate | Regional and moderate losses, difficult to reverse |
|  | Major | Regional and major (or widespread moderate) losses, difficult to reverse |
|  | Massive | Widespread and major losses, irreversible |
| **Impact on ecosystem function** | Minimal concern | Minimal change of function |
|  | Minor | Minor change of function |
|  | Moderate | Moderate change of function |
|  | Major | Major change of function |
|  | Massive | Massive change of all important ecosystem function |

Further detail on the definitions of terms ([Blackburn et al., 2014](#_ENREF_1)):

MC: No effect on fitness of individuals of native species

MI: Causes reductions in individual fitness, but no declines in native population densities

MO: Causes declines in population densities, but no changes in community composition

MR: Causes changes in community composition, that are reversible if the alien species is removed

MV: Causes at least local extinction of species, and irreversible changes in community composition; even if the alien species is removed the system does not recover its original state

We suggested the following as guidelines based on the above definitions:

Minimal concern = small inconsequential changes; 0-10% of species population, habitat or function affected (or lesser impacts on multiple species, habitats or functions)

Minor = changes in size, quality or function of some consequence; 10-25% of species population, habitat or function affected (or lesser impacts on multiple species, habitats or functions)

Moderate = considerable, important changes in size, quality or function; 25-50% of species population, habitat or function affected (or lesser impacts on multiple species, habitats or functions)

Major = large, highly significant changes in size, quality or function; 50-75% of species population, habitat or function affected (or lesser impacts on multiple species, habitats or functions)

Massive = loss of all, or almost all, of a species, function or habitat; 75-100% of species population, habitat or function affected (or lesser impacts on multiple species, habitats or functions)

Confidence scores accompanied by examples to provide context based on the proposed unified framework for environmental impacts ([Blackburn et al., 2014](#_ENREF_1)) and the EPPO Pest Risk Assessment Decision Support Scheme ([EPPO, 2011](#_ENREF_2)).

| **Confidence Score** | **Examples** |
| --- | --- |
| **High** | There is direct relevant evidence to support the assessment.  The situation can easily be predicted.  There are reliable/good quality data sources on impacts of the species.  The interpretation of data/information is straightforward.  Data/information are not controversial, contradictory. |
| **Medium** | There is some evidence to support the assessment.  Some information is indirect, e.g. data from phylogenetically or functionally similar species have been used as supporting evidence.  The interpretation of the data is to some extent ambiguous or contradictory. |
| **Low** | There is no direct evidence to support the assessment, e.g. only data from other species have been used as supporting evidence.  Evidence is poor and difficult to interpret, e.g. because it is strongly ambiguous.  The information sources are considered to be of low quality or contain information that is unreliable. |

**Supporting Information 6: Preliminary species list 1: 249 species listed**

| **ID** | **Subgroup** | **Scientific name** | **English name** |
| --- | --- | --- | --- |
| 1 | Freshwater fish | *Ameiurus catus* | White catfish |
| 2 | Freshwater fish | *Catostomus commersonii* | White sucker |
| 3 | Freshwater fish | *Channa argus* | Northern snakehead |
| 4 | Freshwater fish | *Chrosomus eos (= Phoxinus eos)* | Redbelly dace |
| 5 | Freshwater fish | *Cyprinella lutrensis* | Red shiner |
| 6 | Freshwater fish | *Fundulus heteroclitus* | Mummichog |
| 7 | Freshwater fish | *Gambusia affinis* | Western mosquitofish |
| 8 | Freshwater fish | *Hypostomus plecostomus* | Suckermouth catfish |
| 9 | Freshwater fish | *Micropterus dolomieu* | Smallmouth bass |
| 10 | Freshwater fish | *Misgurnus anguillicaudatus* | Oriental weatherfish |
| 11 | Freshwater fish | *Misgurnus mizolepis* | Chinese weather loach |
| 12 | Freshwater fish | *Monopterus albus* | Swamp eel |
| 13 | Freshwater fish | *Morone americana* | White perch |
| 14 | Freshwater fish | *Oreochromis aureus* | Blue tilapia |
| 15 | Freshwater fish | *Oreochromis mossambicus* | Mossambique tilapia |
| 16 | Freshwater fish | *Oreochromis niloticus* | Nile tilapia |
| 17 | Freshwater fish | *Pimephales promelas* | Fathead minnow |
| 18 | Freshwater fish | *Tilapia mariae* | Spotted tilapia |
| 19 | Freshwater fish | *Tilapia zillii* | Redbelly tilapia |
| 20 | Freshwater fish | *Umbra pygmaea* | Eastern mudminnow |
| 21 | Freshwater invertebrates | *Anopheles quadrimaculatus* | Malaria mosquito |
| 22 | Freshwater invertebrates | *Argulus japonicus* | Japanese fishlouse |
| 23 | Freshwater invertebrates | *Bellamya chinensis* | Chinese mysterysnail |
| 24 | Freshwater invertebrates | *Bellamya japonica* | Japanese mysterysnail |
| 25 | Freshwater invertebrates | *Cherax destructor* | Common yabby |
| 26 | Freshwater invertebrates | *Cherax quadricarinatus* | Redclaw crayfish |
| 27 | Freshwater invertebrates | *Cherax tenuimanus* | Hairy marron |
| 28 | Freshwater invertebrates | *Culex quinquefasciatus* | Southern house mosquito |
| 29 | Freshwater invertebrates | *Cyrtobagous salviniae* | Salvinia weevil |
| 30 | Freshwater invertebrates | *Daphnia lumholtzi* | Water flea |
| 31 | Freshwater invertebrates | *Elimia virginica* | Virginia river snail |
| 32 | Freshwater invertebrates | *Gammarus fasciatus* | Freshwater shrimp |
| 33 | Freshwater invertebrates | *Gillia altilis* | Buffalo pebblesnail |
| 34 | Freshwater invertebrates | *Lasmigonia subviridis* | Green floater |
| 35 | Freshwater invertebrates | *Limnoperna fortunei* | Golden mussel |
| 36 | Freshwater invertebrates | *Lophodella carteri* | Bryozoan |
| 37 | Freshwater invertebrates | *Marissa cornuarietis* | South American giant ramshorn snail |
| 38 | Freshwater invertebrates | *Melanoides tuberculatus* | Red-rim melania |
| 39 | Freshwater invertebrates | *Orconectes obscurus* | Allegheny crayfish |
| 40 | Freshwater invertebrates | *Orconectes rusticus* | Rusty crayfish |
| 41 | Freshwater invertebrates | *Orconectes virilis* | Virile crayfish |
| 42 | Freshwater invertebrates | *Pomacea canaliculata* | Golden apple snail |
| 43 | Freshwater invertebrates | *Pomacea maculata* | Giant apple snail |
| 44 | Freshwater invertebrates | *Procambarus fallax forma virginalis* | Marmokrebs |
| 45 | Freshwater invertebrates | *Skistodiaptomus pallidus* | Copepod |
| 46 | Freshwater invertebrates | *Viviparus georgianus* | Banded mysterysnail |
| 47 | Marine | *Acanthophora spicifera* | A red alga |
| 48 | Marine | *Ascidia sydneiensis* | Green tube tunicate |
| 49 | Marine | *Aulacomya atra* | Bivalve |
| 50 | Marine | *Avrainvillea amadelpha* | A green alga |
| 51 | Marine | *Balanus glandula* | Acorn barnacle |
| 52 | Marine | *Batillaria attramentaria* | Asian horn snail |
| 53 | Marine | *Botrylloides giganteum* | A tunicate |
| 54 | Marine | *Celleporaria brunnea* | A bryozoan |
| 55 | Marine | *Charybdis japonica* | Asian paddle crab |
| 56 | Marine | *Choromytilus chorus* | Bivalve |
| 57 | Marine | *Ciona savignyi* | Pacific transparent tunicate |
| 58 | Marine | *Codium parvulum* | A green alga |
| 59 | Marine | *Crepidula onyx* | Onyx slippersnail |
| 60 | Marine | *Dictyosphaeria cavernosa* | Green bubble weed |
| 61 | Marine | *Didemnum perlucidum* | A colonial tunicate |
| 62 | Marine | *Distaplia bermudensis* | A tunicate |
| 63 | Marine | *Dorvillea similis* | A polychaete worm |
| 64 | Marine | *Gemma gemma* | Gem clam |
| 65 | Marine | *Gracilaria salicornia* | A red alga |
| 66 | Marine | *Grandidierella japonica* | Amphipod |
| 67 | Marine | *Haminoea japonica* | Bubble shell |
| 68 | Marine | *Homarus americanus* | American lobster |
| 69 | Marine | *Ilyanassa obsoleta* | Black dog whelk |
| 70 | Marine | *Kappaphycus alvarezii* | Red alga |
| 71 | Marine | *Laonome calida* | A polychaete |
| 72 | Marine | *Megabalanus coccopoma* | Titan barnacle |
| 73 | Marine | *Molgula ficus* | A tunicate |
| 74 | Marine | *Mytilopsis sallei* | Black striped mussel |
| 75 | Marine | *Neomeris annulata* | Fuzzy tip alga, finger alga |
| 76 | Marine | *Notomastus mossambicus* | A polychaete |
| 77 | Marine | *Nuttallia obscurata* | Purple varnish clam |
| 78 | Marine | *Paranthura japonica* | An isopod |
| 79 | Marine | *Perna viridis* | Asian green mussel |
| 80 | Marine | *Perophora multiclathrata* | A tunicate |
| 81 | Marine | *Phallusia nigra* | A tunicate |
| 82 | Marine | *Plotosus lineatus* | Striped eel catfish |
| 83 | Marine | *Polyopes lancifolius* | A red alga |
| 84 | Marine | *Potamocorbula amurensis* | Asian basket clam |
| 85 | Marine | *Prionospio paucipinnulata* | A polychaete |
| 86 | Marine | *Pteria colymbus* | A bivalve |
| 87 | Marine | *Pterois miles* | Devil firefish, Lion fish |
| 88 | Marine | *Pyura praeputialis* | A tunicate |
| 89 | Marine | *Rhodosoma turcicum* | A unitary tunicate |
| 90 | Marine | *Sphaeroma quoianum* | Australasian isopod |
| 91 | Marine | *Symplegma brakenhielmi* | A tunicate |
| 92 | Marine | *Symplegma reptans* | Pillow-like tunicate |
| 93 | Marine | *Tetrapygus niger* | Sea urchin |
| 94 | Marine | *Zostera japonica* | Dwarf eelgrass |
| 95 | Plants | *Albizia lebbeck* | Indian siris |
| 96 | Plants | *Alternanthera philoxeroides* | Alligator-weed |
| 97 | Plants | *Andropogon virginicius* | Broom-sedge |
| 98 | Plants | *Celastrus orbiculatus* | Oriental bittersweet |
| 99 | Plants | *Chromolaena odorata* | Siam weed |
| 100 | Plants | *Cinnamomum camphora* | Camphor tree |
| 101 | Plants | *Clematis terniflora* | Leather leaf clematis |
| 102 | Plants | *Cortaderia jubata* | Purple pampas grass |
| 103 | Plants | *Cryptostegia grandiflora* | Rubber vine |
| 104 | Plants | *Ehrharta calycina* | Perennial veldtgrass |
| 105 | Plants | *Euonymus fortunei* | Winter creeper |
| 106 | Plants | *Euonymus japonicus* | Japanese spindle |
| 107 | Plants | *Gymnocoronis spilanthoides* | Senegal tea |
| 108 | Plants | *Lespedeza juncea ssp. sericea (= L. cuneata)* | Chinese lespedeza |
| 109 | Plants | *Ligustrum sinense* | Chinese privet |
| 110 | Plants | *Lonicera maackii* | Amur honeysuckle |
| 111 | Plants | *Lonicera morrowii* | Morrow's honeysuckle |
| 112 | Plants | *Lygodium japonicum* | Japanese climbing fern |
| 113 | Plants | *Microstegium vimineum* | Nepalese browntop |
| 114 | Plants | *Pinus patula* | Mexican weeping pine |
| 115 | Plants | *Prosopis juliflora* | Prosopis |
| 116 | Plants | *Prunus campanulata* | Bell flower cherry |
| 117 | Plants | *Rubus rosifolius* | Roseleaf bramble |
| 118 | Plants | *Triadica sebifera (Sapium sebiferum)* | Chinese tallowtree |
| 119 | Plants | *Wedelia trilobata (= Sphagneticola trilobata)* | Wedelia |
| 120 | Terrestrial invertebrates | *Achatina achatina* | Giant ghana snail |
| 121 | Terrestrial invertebrates | *Adelges tsugae* | Hemlock woolly adelgid |
| 122 | Terrestrial invertebrates | *Aeolesthes sarta* | City longhorn beetle, Qetta borer |
| 123 | Terrestrial invertebrates | *Agrilus anxius* | Bronze birch borer |
| 124 | Terrestrial invertebrates | *Agrilus auroguttatus* | Goldspotted oak borer |
| 125 | Terrestrial invertebrates | *Agrilus planipennis* | Emerald ash borer |
| 126 | Terrestrial invertebrates | *Amynthas agrestis* | Crazy snake worm |
| 127 | Terrestrial invertebrates | *Archachatina marginata* | Giant West African snail |
| 128 | Terrestrial invertebrates | *Arthurdendyus triangulatus* | New Zealand flatworm |
| 129 | Terrestrial invertebrates | *Ashworthius sidemi* |  |
| 130 | Terrestrial invertebrates | *Bradybaena similaris* | Asian trampsnail |
| 131 | Terrestrial invertebrates | *Coptotermes formosanus* | Formosan subterranean termite |
| 132 | Terrestrial invertebrates | *Crypticerya genistae* |  |
| 133 | Terrestrial invertebrates | *Culex quinquefasciatus* | Southern house mosquito |
| 134 | Terrestrial invertebrates | *Dendroctonus ponderosae* | Mountain pine beetle |
| 135 | Terrestrial invertebrates | *Dendroctonus rufipennis* | Spruce beetle |
| 136 | Terrestrial invertebrates | *Dendroctonus valens* | Red Turpentine bark beetle |
| 137 | Terrestrial invertebrates | *Dendrolimus sibiricus* | Siberian silk moth |
| 138 | Terrestrial invertebrates | *Dendrolimus superans* | White-lined silk moth |
| 139 | Terrestrial invertebrates | *Diaphorina citri* | Asian citrus psyllid |
| 140 | Terrestrial invertebrates | *Hylobitelus xiaoi* | Chinese large pine weevil |
| 141 | Terrestrial invertebrates | *Latrodectus geometricus* | Black widow spider |
| 142 | Terrestrial invertebrates | *Latrodectus hasselti* | Black widow spider |
| 143 | Terrestrial invertebrates | *Latrodectus mactans* | Black widow spider |
| 144 | Terrestrial invertebrates | *Limicolaria aurora* | Nigerian land snail |
| 145 | Terrestrial invertebrates | *Lissachatina fulica* | Giant African snail |
| 146 | Terrestrial invertebrates | *Malacosoma disstria* | Forest tent caterpillar |
| 147 | Terrestrial invertebrates | *Pachycondyla chinensis* | Asian needle ant |
| 148 | Terrestrial invertebrates | *Pheidole megacephala* | Big-headed ant |
| 149 | Terrestrial invertebrates | *Phoneutria fera* | Brazilian wandering spider |
| 150 | Terrestrial invertebrates | *Platypus quercivorus* | Oak ambrosia beetle |
| 151 | Terrestrial invertebrates | *Polistes chinensis antennalis* | Asian paper wasp |
| 152 | Terrestrial invertebrates | *Polygraphus proximus* | Sakhalin-fir bark beetle |
| 153 | Terrestrial invertebrates | *Saperda candida* | Round-headed apple tree borer |
| 154 | Terrestrial invertebrates | *Scolytus schevyrewi* | Banded elm bark beetle |
| 155 | Terrestrial invertebrates | *Sirex ermak* | Blue-black horntail |
| 156 | Terrestrial invertebrates | *Solenopsis geminata* | Fire ant |
| 157 | Terrestrial invertebrates | *Solenopsis invicta* | Red imported fire ant |
| 158 | Terrestrial invertebrates | *Solenopsis richteri* | Black imported fire ant |
| 159 | Terrestrial invertebrates | *Tetropium gracilicorne* | Fine-horned spruce beetle |
| 160 | Terrestrial invertebrates | *Vespula pensylvanica* | Western yellowjacket |
| 161 | Terrestrial invertebrates | *Wasmannia auropunctata* | Little fire ant |
| 162 | Terrestrial invertebrates | *Xylosandrus mutilatus* | Camphor shoot beetle |
| 163 | Vertebrate | *Acridotheres cristatellus* | Crested myna |
| 164 | Vertebrate | *Acridotheres tristis* | Common myna |
| 165 | Vertebrate | *Amadina fasciata* | Cut-throat |
| 166 | Vertebrate | *Amandava amandava* | Red avadavat |
| 167 | Vertebrate | *Amazona oratrix* | Yellow-headed amazon |
| 168 | Vertebrate | *Ammotragus lervia* | Aoudad |
| 169 | Vertebrate | *Anolis carolinensis* | Carolina anole |
| 170 | Vertebrate | *Anolis sagrei* | Brown anole |
| 171 | Vertebrate | *Anser cygnoides* | Swan goose |
| 172 | Vertebrate | *Axis axis* | Indian spotted deer |
| 173 | Vertebrate | *Bison bison* | American bison |
| 174 | Vertebrate | *Boa constrictor* | Boa constrictor |
| 175 | Vertebrate | *Boiga irregularis* | Brown tree snake |
| 176 | Vertebrate | *Bufo mauritanicus* | Berber toad |
| 177 | Vertebrate | *Callithrix geoffroyi* | White-headed marmoset |
| 178 | Vertebrate | *Callithrix jacchus* | Common marmoset |
| 179 | Vertebrate | *Callithrix penicillata* | Black-tufted marmoset |
| 180 | Vertebrate | *Callosciurus erythraeus* | Pallas's squirrel |
| 181 | Vertebrate | *Callosciurus finlaysonii* | Finlayson's squirrel |
| 182 | Vertebrate | *Camelus dromedarius* | Dromedary |
| 183 | Vertebrate | *Castor canadensis* | American beaver |
| 184 | Vertebrate | *Cercopithecus mona* | Mona monkey |
| 185 | Vertebrate | *Cervus nippon* | Sika deer |
| 186 | Vertebrate | *Chamaeleo jacksonii* | Jackson's chameleon |
| 187 | Vertebrate | *Chelydra serpentina* | Common snapping turtle |
| 188 | Vertebrate | *Chloephaga picta* | Upland goose |
| 189 | Vertebrate | *Chrysemys picta* | Painted turtle |
| 190 | Vertebrate | *Corvus splendens* | House crow |
| 191 | Vertebrate | *Ctenosaura similis* | Black iguana |
| 192 | Vertebrate | *Cynops pyrrhogaster* | Japanese fire-bellied salamander |
| 193 | Vertebrate | *Elaphe guttata* | Corn snake |
| 194 | Vertebrate | *Eleutherodactylus coqui* | Common coqui |
| 195 | Vertebrate | *Eleutherodactylus planirostris* | Greenhouse frog |
| 196 | Vertebrate | *Estrilda astrild* | Common waxbill |
| 197 | Vertebrate | *Estrilda melpoda* | Orange-cheeked waxbill |
| 198 | Vertebrate | *Estrilda troglodytes* | Black-rumped waxbill |
| 199 | Vertebrate | *Euplectes afer* | Yellow-crowned bishop |
| 200 | Vertebrate | *Felis bengalensis* | Leopard cat |
| 201 | Vertebrate | *Gecko gecko* | Tokay gecko |
| 202 | Vertebrate | *Graptemys geographica* | Northern map turtle |
| 203 | Vertebrate | *Graptemys pseudogeographica* | False map turtle |
| 204 | Vertebrate | *Gymnorhina tibicen* | Australian magpie |
| 205 | Vertebrate | *Hemidactylus frenatus* | Common house gecko |
| 206 | Vertebrate | *Herpestes auropunctatus* | Small Asian mongoose |
| 207 | Vertebrate | *Hydrochoerus hydrochoeris* | Capybara |
| 208 | Vertebrate | *Iguana iguana* | Green iguana |
| 209 | Vertebrate | *Lampropeltis getula* | Common kingsnake |
| 210 | Vertebrate | *Leiothrix lutea* | Red-billed leiothrix |
| 211 | Vertebrate | *Lonchura malabarica* | White-throated munia |
| 212 | Vertebrate | *Macrochelys temminckii* | Aligator snapping turtle |
| 213 | Vertebrate | *Muntiacus reevesi* | Reeve's muntjac |
| 214 | Vertebrate | *Nandayus nenday* | Nanday parakeet |
| 215 | Vertebrate | *Nasua nasua* | Coati |
| 216 | Vertebrate | *Numida meleagris* | Helmeted guineafowl |
| 217 | Vertebrate | *Nymphicus hollandicus* | Cockatiel |
| 218 | Vertebrate | *Paradoxornis alphonsianus* | Ashy-throated parrotbill |
| 219 | Vertebrate | *Paradoxornis webbianus* | Vinous-throated parrotbill |
| 220 | Vertebrate | *Pelodiscus sinensis* | Chinese softshell turtle |
| 221 | Vertebrate | *Peromyscus fraterculus* | Northern Baja deermouse |
| 222 | Vertebrate | *Petrogale inornata* | Unadorned rock-wallaby |
| 223 | Vertebrate | *Phoenicopterus chilensis* | Chilean flamingo |
| 224 | Vertebrate | *Pitangus sulphuratus* | Great kiskadee |
| 225 | Vertebrate | *Ploceus galbula* | Rüppell's weaver |
| 226 | Vertebrate | *Ploceus melanocephalus* | Black-headed weaver |
| 227 | Vertebrate | *Poicephalus senegalus* | Senegal parrot |
| 228 | Vertebrate | *Pseudemys concinna* | River cooter |
| 229 | Vertebrate | *Psittacara acuticaudatus* | Blue-crowned parakeet |
| 230 | Vertebrate | *Psittacara erythrogenys* | Red-masked parakeet |
| 231 | Vertebrate | *Psittacara mitratus* | Mitred parakeet |
| 232 | Vertebrate | *Psittacula eupatria* | Alexandrine parakeet |
| 233 | Vertebrate | *Pycnonotus cafer* | Red-vented bulbul |
| 234 | Vertebrate | *Pycnonotus jocosus* | Red-whiskered bulbul |
| 235 | Vertebrate | *Python molurus* | Indian rock python |
| 236 | Vertebrate | *Quelea quelea* | Red-billed quelea |
| 237 | Vertebrate | *Rhea americana* | Greater rhea |
| 238 | Vertebrate | *Rhinella marina* | Cane toad |
| 239 | Vertebrate | *Sciurus niger* | Fox squirrel |
| 240 | Vertebrate | *Streptopelia roseogrisea* | African collared-dove |
| 241 | Vertebrate | *Sylvilagus floridanus* | Eastern cottontail |
| 242 | Vertebrate | *Sylvilagus transitionalis* | N England cottontail |
| 243 | Vertebrate | *Tamiasciurus hudsonicus* | American red squirrel |
| 244 | Vertebrate | *Tenrec ecaudatus* | Common tenrec |
| 245 | Vertebrate | *Testudo horsfieldii* | Russian tortoise |
| 246 | Vertebrate | *Trichosurus vulpecula* | Brushtail possum |
| 247 | Vertebrate | *Vidua macroura* | Pin-tailed wydah |
| 248 | Vertebrate | *Xenopus laevis* | African clawed frog |
| 249 | Vertebrate | *Zosterops japonicus* | Japanese white-eye |

**Supporting Information 7: Preliminary species list 2: 120 species listed**

| **ID** | **Rank** | **Subgroup** | **Scientific name** | **English Name** |
| --- | --- | --- | --- | --- |
| 1 | VERY HIGH | Freshwater | *Channa argus* | Northern snakehead |
| 2 | VERY HIGH | Freshwater | *Gambusia affinis* | Western mosquitofish |
| 3 | VERY HIGH | Freshwater | *Limnoperna fortunei* | Golden mussel |
| 4 | VERY HIGH | Freshwater | *Micropterus dolomieu* | Smallmouth bass |
| 5 | VERY HIGH | Freshwater | *Orconectes rusticus* | Rusty crayfish |
| 6 | VERY HIGH | Freshwater | *Orconectes virilis* | Virile crayfish |
| 7 | VERY HIGH | Freshwater | *Oreochromis aureus* | Blue tilapia |
| 8 | VERY HIGH | Freshwater | *Oreochromis mossambicus* | Mossambique tilapia |
| 9 | VERY HIGH | Freshwater | *Oreochromis niloticus* | Nile tilapia |
| 10 | VERY HIGH | Freshwater | *Pomacea canaliculata* | Golden apple snail |
| 11 | VERY HIGH | Freshwater | *Pomacea maculata* | Giant apple snail |
| 12 | VERY HIGH | Marine | *Botrylloides giganteum* | Tunicate |
| 13 | VERY HIGH | Marine | *Codium parvulum* | A green alga |
| 14 | VERY HIGH | Marine | *Crepidula onyx* | Onyx slippersnail |
| 15 | VERY HIGH | Marine | *Homarus americanus* | American Lobster |
| 16 | VERY HIGH | Marine | *Mytilopsis sallei* | Black striped mussel |
| 17 | VERY HIGH | Marine | *Penaeus aztecus* | Northern brown shrimp |
| 18 | VERY HIGH | Marine | *Plotosus lineatus* | Striped eel catfish |
| 19 | VERY HIGH | Marine | *Pterois miles* | Devil firefish, Lion fish |
| 20 | VERY HIGH | Plants | *Alternanthera philoxeroides* | Alligator-weed |
| 21 | VERY HIGH | Terr_Inverts | *Arthurdendyus triangulatus* | New Zealand flatworm |
| 22 | VERY HIGH | Vertebrates | *Acridotheres tristis* | Common myna |
| 23 | VERY HIGH | Vertebrates | *Bufo mauritanicus* | Berber toad |
| 24 | VERY HIGH | Vertebrates | *Callosciurus erythraeus* | Pallas's squirrel |
| 25 | VERY HIGH | Vertebrates | *Callosciurus finlaysonii* | Finlayson's squirrel |
| 26 | VERY HIGH | Vertebrates | *Corvus splendens* | House Crow |
| 27 | VERY HIGH | Vertebrates | *Herpestes auropunctatus* | Small Asian mongoose |
| 28 | VERY HIGH | Vertebrates | *Lampropeltis getula* | Common kingsnake |
| 29 | VERY HIGH | Vertebrates | *Nasua nasua* | Coati |
| 30 | VERY HIGH | Vertebrates | *Pycnonotus cafer* | Red-vented bulbul |
| 31 | VERY HIGH | Vertebrates | *Sciurus niger* | Fox squirrel |
| 32 | HIGH | Freshwater | *Bellamya chinensis* | Chinese mysterysnail |
| 33 | HIGH | Freshwater | *Cherax destructor* | Common yabby |
| 34 | HIGH | Freshwater | *Cherax quadricarinatus* | Redclaw crayfish |
| 35 | HIGH | Freshwater | *Cyprinella lutrensis* | Red shiner |
| 36 | HIGH | Freshwater | *Fundulus heteroclitus* | Mummichog |
| 37 | HIGH | Freshwater | *Gammarus fasciatus* | Freshwater shrimp |
| 38 | HIGH | Freshwater | *Hypostomus plecostomus* | Suckermouth catfish |
| 39 | HIGH | Freshwater | *Marissa cornuarietis* | South American giant ramshorn snail |
| 40 | HIGH | Freshwater | *Misgurnus anguillicaudatus* | Oriental weatherfish |
| 41 | HIGH | Freshwater | *Misgurnus mizolepis* | Chinese weather loach |
| 42 | HIGH | Freshwater | *Morone americana* | White perch |
| 43 | HIGH | Freshwater | *Tilapia zillii* | Redbelly tilapia |
| 44 | HIGH | Marine | *Acanthophora spicifera* | A red alga |
| 45 | HIGH | Marine | *Charybdis japonica* | Asian paddle crab |
| 46 | HIGH | Marine | *Macrorhynchia philippina* | White stinger |
| 47 | HIGH | Marine | *Perna viridis* | Asian green mussel |
| 48 | HIGH | Marine | *Potamocorbula amurensis* | Asian basket clam |
| 49 | HIGH | Marine | *Pseudonereis anomala* | A polychaete |
| 50 | HIGH | Marine | *Symplegma reptans* | Pillow-like tunicate |
| 51 | HIGH | Plants | *Albizia lebbeck* | Indian siris |
| 52 | HIGH | Plants | *Andropogon virginicus* | Broom-sedge |
| 53 | HIGH | Plants | *Celastrus orbiculatus* | Oriental bittersweet |
| 54 | HIGH | Plants | *Chromolaena odorata* | Siam weed |
| 55 | HIGH | Plants | *Cinnamomum camphora* | Camphor tree |
| 56 | HIGH | Plants | *Clematis terniflora* | Leather leaf clematis |
| 57 | HIGH | Plants | *Cortaderia jubata* | Purple pampas grass |
| 58 | HIGH | Plants | *Cryptostegia grandiflora* | Rubber vine |
| 59 | HIGH | Plants | *Ehrharta calycina* | Perennial veldtgrass |
| 60 | HIGH | Plants | *Euonymus fortunei* | Winter creeper |
| 61 | HIGH | Plants | *Euonymus japonicus* | Japanese spindle |
| 62 | HIGH | Plants | *Gymnocoronis spilanthoides* | Senegal tea |
| 63 | HIGH | Plants | *Lespedeza juncea ssp. sericea (= L. cuneata)* | Chinese lespedeza |
| 64 | HIGH | Plants | *Ligustrum sinense* | Chinese privet |
| 65 | HIGH | Plants | *Lonicera maackii* | Amur honeysuckle |
| 66 | HIGH | Plants | *Lonicera morrowii* | Morrow's honeysuckle |
| 67 | HIGH | Plants | *Lygodium japonicum* | Japanese climbing fern |
| 68 | HIGH | Plants | *Microstegium vimineum* | Nepalese browntop |
| 69 | HIGH | Plants | *Prosopis juliflora* | Prosopis |
| 70 | HIGH | Plants | *Prunus campanulata* | Bell flower cherry |
| 71 | HIGH | Plants | *Rubus rosifolius* | Roseleaf bramble |
| 72 | HIGH | Plants | *Triadica sebifera (Sapium sebiferum)* | Chinese tallowtree |
| 73 | HIGH | Plants | *Wedelia trilobata (= Sphagneticola trilobata)* | Wedelia |
| 74 | HIGH | Terr_Inverts | *Aeolesthes sarta* | City longhorn beetle, Qetta borer |
| 75 | HIGH | Terr_Inverts | *Amynthas agrestis* | Crazy snake worm |
| 76 | HIGH | Terr_Inverts | *Ashworthius sidemi* | - |
| 77 | HIGH | Terr_Inverts | *Pachycondyla chinensis* | Asian needle ant |
| 78 | HIGH | Terr_Inverts | *Pheidole megacephala* | Big-headed ant |
| 79 | HIGH | Terr_Inverts | *Saperda candida* | Round-headed apple tree borer |
| 80 | HIGH | Terr_Inverts | *Sirex ermak* | Blue-black horntail |
| 81 | HIGH | Terr_Inverts | *Solenopsis geminata* | Fire ant |
| 82 | HIGH | Terr_Inverts | *Solenopsis invicta* | Red imported fire ant |
| 83 | HIGH | Terr_Inverts | *Solenopsis richteri* | Black imported fire ant |
| 84 | HIGH | Terr_Inverts | *Tetropium gracilicorne* | Fine-horned spruce beetle |
| 85 | HIGH | Terr_Inverts | *Vespula pensylvanica* | Western yellowjacket |
| 86 | HIGH | Vertebrates | *Acridotheres cristatellus* | Crested myna |
| 87 | HIGH | Vertebrates | *Axis axis* | Indian spotted deer |
| 88 | HIGH | Vertebrates | *Bison bison* | American bison |
| 89 | HIGH | Vertebrates | *Boiga irregularis* | Brown tree snake |
| 90 | HIGH | Vertebrates | *Chrysemys picta* | Painted turtle |
| 91 | HIGH | Vertebrates | *Cynops pyrrhogaster* | Japanese fire-bellied salamander |
| 92 | HIGH | Vertebrates | *Eleutherodactylus coqui* | Common coquí |
| 93 | HIGH | Vertebrates | *Eleutherodactylus planirostris* | Greenhouse frog |
| 94 | HIGH | Vertebrates | *Hemidactylus frenatus* | Common house gecko |
| 95 | HIGH | Vertebrates | *Psittacula eupatria* | Alexandrine parakeet |
| 96 | HIGH | Vertebrates | *Pycnonotus jocosus* | Red-whiskered bulbul |
| 97 | HIGH | Vertebrates | *Rhea americana* | Greater rhea |
| 98 | HIGH | Vertebrates | *Rhinella marina* | Cane toad |
| 99 | HIGH | Vertebrates | *Trichosurus vulpecula* | Brushtail possum |
| 100 | MEDIUM | Freshwater | *Culex quinquefasciatus* | Southern house mosquito |
| 101 | MEDIUM | Freshwater | *Daphnia lumholtzi* | Water flea |
| 102 | MEDIUM | Marine | *Ascidia sydneiensis* | Green tube tunicate |
| 103 | MEDIUM | Marine | *Balanus glandula* | Acorn Barnacle |
| 104 | MEDIUM | Marine | *Ciona savignyi* | Pacific transparent tunicate |
| 105 | MEDIUM | Marine | *Dictyosphaeria cavernosa* | Green bubble weed |
| 106 | MEDIUM | Marine | *Didemnum perlucidum* | A colonial tunicate |
| 107 | MEDIUM | Marine | *Dorvillea similis* | A polychaete worm |
| 108 | MEDIUM | Marine | *Polyopes lancifolius* | A red alga |
| 109 | MEDIUM | Marine | *Rhodosoma turcicum* | A unitary tunicate |
| 110 | MEDIUM | Marine | *Zostera japonica* | Dwarf eelgrass |
| 111 | MEDIUM | Plants | *Pinus patula* | Mexican weeping pine |
| 112 | MEDIUM | Terr_Inverts | *Agrilus auroguttatus* | Goldspotted oak borer |
| 113 | MEDIUM | Terr_Inverts | *Dendrolimus superans* | White-lined silk moth |
| 114 | MEDIUM | Terr_Inverts | *Megaplatypus mutatus* | Grand forest borer |
| 115 | MEDIUM | Terr_Inverts | *Platypus quercivorus* | Oak ambrosia beetle |
| 116 | MEDIUM | Vertebrates | *Boa constrictor* | Boa constrictor |
| 117 | MEDIUM | Vertebrates | *Gymnorhina tibicen* | Australian magpie |
| 118 | MEDIUM | Vertebrates | *Python molurus* | Indian rock python |
| 119 | MEDIUM | Vertebrates | *Quelea quelea* | Red-billed quelea |
| 120 | MEDIUM | Vertebrates | *Tamiasciurus hudsonicus* | American red squirrel |

**Supporting Information 8: Tables and codes**

Functional groups and associated codes used in the compilation of information on IAS for consideration within the horizon scanning

| Functional group | Code |
| --- | --- |
| Detritivore | Det |
| Primary producer | PP |
| Filter feeder | Filter |
| Herbivore | Herb |
| Predator or parasite | Pred |
| Omnivore | Omni |
| Pollinator | Poll |

Native distributions (geographic region) for terrestrial and freshwater species and associated codes used in the compilation of information on IAS for consideration within the horizon scanning

| Geographic region | Code |
| --- | --- |
| Europe | Eur |
| Africa | Afr |
| Asia-temperate | As |
| Asia-tropical | At |
| Australasia | Aus |
| Pacific | Pac |
| N America | NAm |
| S America | SAm |
| Antarctica | Ant |

Broad biogeographic groups modified from the EEA regions and applied to the marine species in relation to likely bioregions to be affected by the arrival, establishment, spread and impact of the alien species within the next ten years

| Code | Bioregion |  |
| --- | --- | --- |
| MAC | Macaronesia | Canary Islands, Madeira, Azores |
| MED | Mediterranean |  |
| BLK | Black Sea |  |
| ATL | NE Atlantic |  |
| BAL | Baltic |  |

Global biogeographic regions applied to the marine species in relation to native range and invaded areas outside of Europe modified from Spalding (2007) <http://www.nature.org/ourinitiatives/regions/northamerica/unitedstates/colorado/scienceandstrategy/marine-ecoregions-of-the-world.pdf>

| Code | Bioregion |  |
| --- | --- | --- |
| ARC | Arctic | Alaska, N Canada, N Russia |
| TeNWP | Temperate NW Pacific | Japan, Korea, N China, E Russia |
| TeNEP | Temperate NE Pacific | W Canada, W USA (California northwards), S Alaska |
| TeNWA | Temperate NW Atlantic | E USA, E Canada |
| TeNEA | Temperate NE Atlantic | Europe, NW Africa |
| EIP | Eastern Indo-Pacific | Hawaii, Guam |
| CIP | Central Indo-Pacific | Philippines, Malaysia, Taiwan, N Australia |
| WIP | Western Indo-Pacific | India, E Africa, Red Sea |
| TrEP | Tropical Eastern Pacific | Central America |
| TrEA | Tropical Eastern Atlantic | W Africa |
| TrWA | Tropical Western Atlantic | Caribbean, Brazil |
| TeSEP | Temperate SE Pacific | Chile, Peru |
| TeSWA | Temperate SW Atlantic | Argentina |
| TeSAf | Temperate Southern Africa | S Africa, Namibia |
| TeAu | Temperate Australasia | Australia, NZ |
| SOU | Southern Ocean | Antarctica |

Potential pathways through which IAS could arrive were classified according to the scheme outlined by the CBD ([CBD 2014](#_ENREF_18)). Multiple pathways are relevant for many species and these were documented as a list.

| Category | Subcategory | Code |
| --- | --- | --- |
| Release in nature | Biological Control  Erosion control / dune stabilisation (windbreaks/hedges)  Fishery in the wild  Hunting  Landscape/flora/fauna improvement in the wild  Introduction for conservation purposes or wildlife management  Release in nature for use (other than above)  Other intentional release | BC  EC  F  H  L  Cons  R  Other |
| Escape from confinement | Agriculture  Aquaculture  Botanical garden/zoo/aquaria  Pet/aquarium/terranium  Farmed animals  Forestry  Fur Farm  Hortiulture  Ornamental other than horticulture  Research  Live food and live bait  Other escape from confinement | Ag  Aq  BZA  Pet  Farm  For  FF  Hort  Orn  Res  Live  Other escape |
| Transport contaminant | Contaminant nursery material  Contaminated bait  Food contaminant  Contaminant on animals (except parasites)  Parasites on animals  Contaminant on plants (except parasites)  Parasites on plants  Seed contaminant  Timber trade  Transportation of habitat material | CNM  Bait  Food  Con Anim  Par Anim  Con Plant  Par Plant  Seed  TT  THM |
| Transport-stowaway | Angling/fishing equipment  Container/bulk  Hitchhikers on airplane  Hitchhikers on ship/boat  Machinery/equipment  People and luggage / equipment  Organic packing material  Ship/boat ballast water  Ship/boat hull fouling  Vehicles  Other means of transport | Ang  Container  Air  Ship  Mach  Lug  Org  Ballast  Hull  Veh  Other transport |
| Corridor | Interconnected waterways – Water Tunnels and bridges | Tun |
| Unaided | Natural dispersal across border of IAS that have been introduced through pathways 1-5 | Nat |

**Supplementary References**

Dullinger, I., Wessely, J., Bosdorf, O., Dawson, W., Essl, F., Gattringer, A., Klonner, G., Moser, D., Kuttner, M., Pergl, P., Pyšek, P., Thuiller, W., van Kleunen, M., Weigelt, P., Winter, M. & Dullinger, S. (2017). Global change will increase the naturalization risk from garden plants in Europe. *Global Change Biology*, 26, 43–53.

EPPO (2011). Decision-support scheme for quarantine pests. http://archives.eppo.int/EPPOStandards/PM5_PRA/PRA_scheme_2011.pdf.

Randall, R.P. (2007). The introduced flora of Australia and its weed status. CRC for Australian Weed Management, Glen Osmond.

Faulkner, K.T., Robertson M.P., Rouget M. & Wilson, J.R.U. (2014). A simple, rapid methodology for developing invasive species watch lists. *Biological Conservation*, 179, 25–32.

Pheloung, P.C., Williams, P.A. & Halloy, S.R. (1999) A weed risk assessment model for use as a biosecurity tool evaluating plant introductions. *Journal of Environmental Management*, 57, 239–251.

Weber, E. & Gut D. (2004). Assessing the risk of potentially invasive plant species in central Europe. *Journal for Nature Conservation*, 12, 171–179.
